# Gait analysis patterns and rehabilitative interventions to improve gait in persons with hereditary spastic paraplegia: a systematic review and meta-analysis

**DOI:** 10.3389/fneur.2023.1256392

**Published:** 2023-09-20

**Authors:** Silvia Faccioli, Angela Cavalagli, Nicola Falocci, Giulia Mangano, Irene Sanfilippo, Silvia Sassi

**Affiliations:** ^1^Children Rehabilitation Unit, Azienda Unità Sanitaria Locale IRCCS di Reggio Emilia, Reggio Emilia, Italy; ^2^Clinical and Experimental Medicine, Department of Biomedical, Metabolic and Neural Sciences, University of Modena and Reggio Emilia, Modena, Italy; ^3^Children Rehabilitation Unit, IRCCS Fondazione Don Carlo Gnocchi, Milano, Italy; ^4^Office of Policy Evaluation and Statistical Studies, Umbria Legislative Assembly, Perugia, Italy; ^5^Department of Physical Medicine and Rehabilitation, Azienda Sanitaria Provinciale 3 (ASP 3), Acireale Hospital, Catania, Italy; ^6^Rehabilitation Center CMR, Adrano, Catania, Italy

**Keywords:** gait analysis, walking, physical therapy modalities, rehabilitation, spasticity, botulinum toxins, spastic paraparesis, gait disorders

## Abstract

**Background:**

Hereditary spastic paraplegias (HSPs) are a group of inheritance diseases resulting in gait abnormalities, which may be detected using instrumented gait analysis. The aim of this systematic review was 2-fold: to identify specific gait analysis patterns and interventions improving gait in HSP subjects.

**Methods:**

A systematic review was conducted in PubMed, Cochrane Library, REHABDATA, and PEDro databases, in accordance with reporting guidelines of PRISMA statement and Cochrane's recommendation. The review protocol was recorded on the PROSPERO register. Patients with pure and complicated HSP of any age were included. All types of studies were included. Risk of bias, quality assessment, and meta-analysis were performed.

**Results:**

Forty-two studies were included: 19 were related to gait analysis patterns, and 24 were intervention studies. The latter ones were limited to adults. HSP gait patterns were similar to cerebral palsy in younger subjects and stroke in adults. Knee hyperextension, reduced range of motion at knee, ankle, and hip, reduced foot lift, and increased rapid trunk and arm movements were reported. Botulinum injections reduced spasticity but uncovered weakness and improved gait velocity at follow-up. Weak evidence supported intrathecal baclofen, active intensive physical therapy (i.e., robot-assisted gait training, functional exercises, and hydrotherapy), and functional electrical stimulation. Some improvements but adverse events were reported after transcranial magnetic stimulation, transcutaneous spinal direct current stimulation, and spinal cord stimulation implant.

**Conclusion:**

Knee hyperextension, non-sagittal pelvic movements, and reduced ROM at the knee, ankle, and hip represent the most peculiar patterns in HSP, compared to diplegic cerebral palsy and stroke. Botulinum improved comfortable gait velocity after 2 months. Nonetheless, interventions reducing spasticity might result in ineffective functional outcomes unveiling weakness. Intensive active physical therapy and FES might improve gait velocity in the very short term.

## 1. Introduction

Hereditary spastic paraplegia (HSP) is a heterogeneous and large group of neurodegenerative diseases of which the main common feature is lower limb spasticity and weakness, based on the retrograde distal degeneration of the corticospinal and posterior column pathways (1). The key diagnostic clinical finding, characterizing the pure forms, is progressive upper motor neuron (UMN) syndrome of the lower limb which includes spasticity (1), hyperreflexia, extensor plantar responses, weakness, and loss of selective control (2, 3). In complicated forms (4), additional neurologic deficits are present, such as ataxia, amyotrophy, optic atrophy, pigmentary retinopathy, intellectual disability, extrapyramidal signs, dementia, deafness, ichthyosis, peripheral neuropathy, and epilepsy, with neuroimaging abnormalities such as cerebellar atrophy (2). Prevalence is estimated at 3–10 cases per 100.000 in the European population (2) and incidence at 1.27–9.6/100.000 (5). Depending on the presence or absence of a family history of spastic paraparesis and the results of genetic testing, the disease is named HSP or SSP, as sporadic (6). The genetic basis of HSP is complex, with more than 70 known subtypes involving autosomal, dominant or recessive, and X-linked inheritance patterns (1, 7), causing dysfunction of protein involved in intracellular trafficking or mitochondrial function (7, 8). The age of symptom onset, rate of progression, and degree of disability are often variable among different genetic types of HSP, as well as within individual families having the same gene mutation (6, 9, 10). Early childhood onset forms tend to be relatively non-progressive over many years, resembling spastic diplegia forms of cerebral palsy (11). On the contrary, late onset is associated with more progressive disease and gait decline (9, 12).

The gait impairment is the most frequent clinical sign in HSP patients, and it is often recognized as the onset symptom (10, 13). It results from the combination of several factors such as spasticity, weakness, loss of selective control, impaired proprioception, and vibratory sensitivity (3). Identifying the gait characteristics and evolution of HSP subjects is the key to develop a gait functional prognosis for this population and formulate appropriate interventions. In addition, differentiating the HSP gait pattern from similar ones, observed in other pathologies, is desirable to support differential diagnosis. Moreover, gait capacity and balance are mutually influenced; then, analyzing the gait pattern is also useful for identifying specific issues that increase the risk of falls (14). Computerized gait analysis (GA) is the best way to provide a reliable and repeatable measurement of specific gait parameters and impairments (6, 15). Some authors have investigated the deficits in gait in HSP using GA, compared to their healthy peers or other patients, mostly to stroke or spastic diplegic cerebral palsy (DCP) subjects. Nonetheless, no review has systematically summarized the evidence from these studies to comprehensively describe the different observed gait patterns. Some review studies have been published (16, 17) regarding treatment in HSP patients, but none focused on the effect of the rehabilitative treatment on gait function. The aim of the present systematic review was 2-fold: to identify which gait patterns characterize HSP patients using computed gait analysis and to identify which rehabilitative treatment (orthotic devices, botulinum toxin, physiotherapy, physical therapy, and other approaches) leads to improvement in any type of gait parameters in hereditary spastic paraplegia patients.

## 2. Methods

### 2.1. Search and selection

The present study consists of a systematic review of primary studies and was performed and reported in accordance with the reporting guidelines of the PRISMA statement (18) and Cochrane's methodological recommendation (19). The review protocol was registered on the PROSPERO public online register for systematic review, with registration number CRD42021290141. The study was conducted according to the pre-specified protocol, except for quality and risk-of-bias assessment, which was performed with more specific and adequate tools; in addition, a meta-analysis was performed.

The scope of the systematic review was structured according to the Patients, Intervention, Control, and Outcome (PICO) framework for intervention:

- P: patients of all ages with a diagnosis of pure or complicated form of HSP
- I: gait pattern description (3D gait analysis) or intervention to improve gait pattern (orthotic devices/botulinum toxin/physiotherapy/physical therapy, and other rehabilitative approaches).
- C: gait analysis pattern of healthy controls or of patients affected by other diseases and/or no intervention or different interventions to improve gait function in HSP
- O: variables of 3-dimensional gait analysis (kinematics and/or kinetics and/or surface electromyography and/or spatiotemporal parameters) for gait pattern description. Any gait parameter or outcome measure to assess gait improvement after intervention (gait analysis and/or walk/gait speed and/or mobility test and/or spatiotemporal parameters and/or any type of walking test).

A unique search strategy was considered including both aims of the present review, based on an overlap of keywords and terms. Search procedures are described in Supplementary Table 1. A literature search was performed on 10 May 2021 in four international databases (PubMed, Cochrane Library, REHABDATA, and PEDro). Articles published from the inception of databases to 10 May 2021 were searched, with no limit relative to the year of publication, language, age, and type of primary study design. Other articles were also obtained from the reference lists of articles identified by the primary search in the databases. Pharmacological treatments were excluded from the search because they were the object of a recent review study (16).

The population of interest included ambulatory HSP patients, able to perform gait analysis, with a definite diagnosis of HSP or HSP/SSP according to Harding or McDermott criteria (13, 20), with both pure and complicated forms, of all ages. Studies were included if they presented a gait analysis evaluation in HSP patients, with or without comparison with healthy or pathological controls. Studies assessing gait function, by means of any type of gait outcome measure, following an intervention, were also included. Outcomes of interest were the variables of computerized gait analysis (including spatiotemporal parameters and kinetic and kinematic variables) and any type of gait assessment only following gait-focused treatment. Exclusion criteria were as follows: animal study, languages different from English and Italian with no possibility to achieve an official translated version, ongoing study or lacking publication of results, and mixed samples without reporting specific results in HSP patients. According to these inclusion and exclusion criteria, all studies were screened first by title/abstract and then by full text by two independent groups of two authors each (SF, AC, GM, and IS). Each group was blind to each other's decisions. Any disagreement was resolved through discussion among authors. Not retrieved articles and ongoing studies were just recorded as not retrieved.

### 2.2. Data extraction

Two authors independently completed the data extraction (SF and AC), sorting the information into two different content areas, one focused on HSP gait-analysis-pattern description and the other focused on rehabilitative interventions to improve gait (intervention). The authors extracted data about study design and methodology, participant characteristics, protocol details, outcome measures, and results of the studies. Any disagreement among the authors was discussed and resolved by consensus.

### 2.3. Quality and risk-of-bias assessment

The quality of studies was assessed by means of a checklist approach using the Joanna Briggs Institute (JBI) critical appraisal tool (21) for case–control studies, case series, and case report studies. These scales enquired 8 to 10 items, questioning information regarding study design, population, intervention, and outcome details, and whenever appropriate, statistical analysis quality. According to the study by George et al. (22), a cutoff score >70% was considered a sufficient level of quality, while a quality score equal or lower suggested some methodological limitations. The National Institutes of Health (NHI) quality assessment tool (23, 24) was used for quality assessment in before-after (pre-post) studies without a control group, assigning a quality rating as “Good”, “Fair”, or “Poor” according to NIH guidance (23). This scale consists of 12 items questioning the studies' internal validity and risk of bias. The Physiotherapy Evidence Database (PEDro) scale (25) was used for randomized controlled studies (RCTs). It consists of 11 items enquiring information about inclusion criteria, randomization and assignation process, population features, blinding of patients and operators, dropout and missing data, results, and statistical analysis report. Total PEDro scores of 0–3 were considered “Poor”, 4–5 “Fair”, 6–8 “Good”, and 9–10 “Excellent”. The risk of bias (RoB) was assessed also with a domain-based approach using the Risk of Bias in Non-randomized Studies-of Interventions (ROBINS-I) (26, 27) tool in controlled studies and using version 2 of the Cochrane risk-of-bias (ROB2) tool for RCTs (specific for crossover design) (28, 29). The ROBINS tool enquired about the following dimensions: bias due to confounding (D1), in the selection of participants (D2) and the classification of interventions (D3), deviation from intended interventions (D4), missing outcome data (D5), bias in the measurement of the outcome (D6), and in the selection of the reported results (D7). The ROB2 tool enquired about the following dimensions: bias arising from the randomization process (D1) and from period and carryover effects (D1b), bias due to deviation from the intended intervention (D2), and to missing outcome data (D3), and bias in the measurement of the outcome (D4) and in the selection of the reported results (D5). The same two independent groups of reviewers (SF, AC, GM, and IS) assessed the methodological quality and the risk of bias of all the included studies. Any disagreement between the two groups was resolved through discussion among the authors. The assessment of quality and RoB did not provide criteria for excluding articles but for stratifying them.

### 2.4. Meta-analysis

Studies performing the same type of treatment, sharing almost one outcome measure, and having a sample >1 participant were selected for the meta-analysis.

The meta-analyses were carried out using R software (30) and the package “Metafor” (31) on the main results of the selected studies that include the number of observations (*n*), the means, and the standard deviations (*sd*). Heterogeneity among the studies was tested with Cochran's *Q*-test (32), which tests whether the variability in the observed effect sizes or outcomes is larger than would be expected based on sampling variability alone. The estimation of the weighted means was carried out via a fixed effect model when no significant heterogeneity was detected among studies, or a random effect model otherwise.

To evaluate the significance of the effect of the treatment at the different time points, a random effect model was used, estimating the standardized mean difference and reporting the 95% confidence interval as summary statistics. The standard deviation of the change was performed with the method suggested by Morris et al. (33), taking the correlation coefficient *r* = 0.40 as a conservative estimate.

Since studies might have differences in such aspects as quality, which might influence the result of meta-analysis, a sensitivity analysis was conducted changing the effect model and removing the studies with a higher risk of bias to confirm the robustness of our findings.

## 3. Results

**Figure 1:**
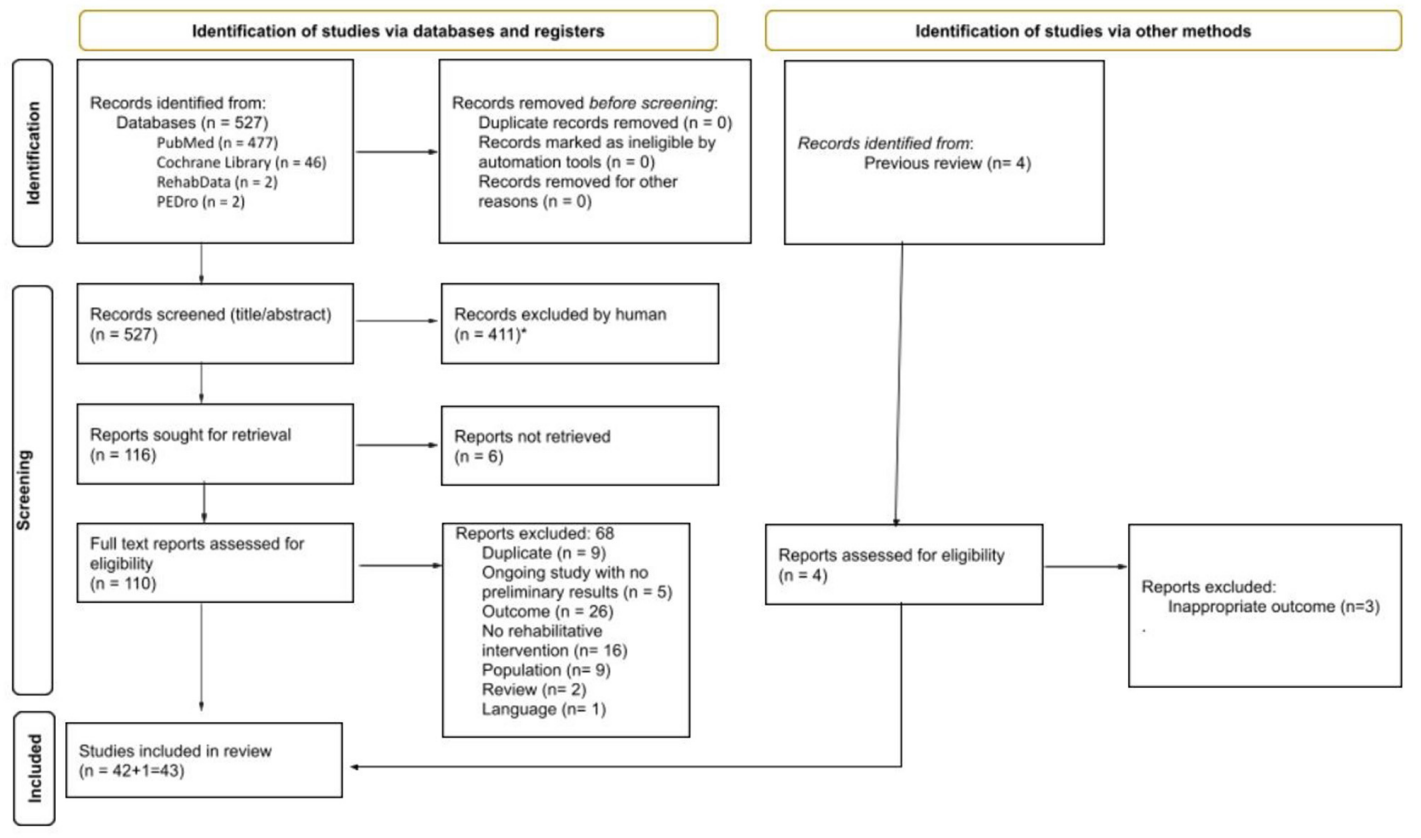
PRISMA flow diagram.

Figure 1 provides details about study identification and selection (PRISMA flow diagram). A total of 527 records were found through database searches. Exclusion based on title/abstract screening resulted in 116 full texts being examined for eligibility, whereas 411 articles did not meet the inclusion criteria. After full-text analysis, 43 studies were finally included in the review and were divided as follows: 19 in the gait analysis pattern (GA pattern) database and 24 in the intervention database.

### 3.1. Quality and risk-of-bias assessment

**Figure 2:**
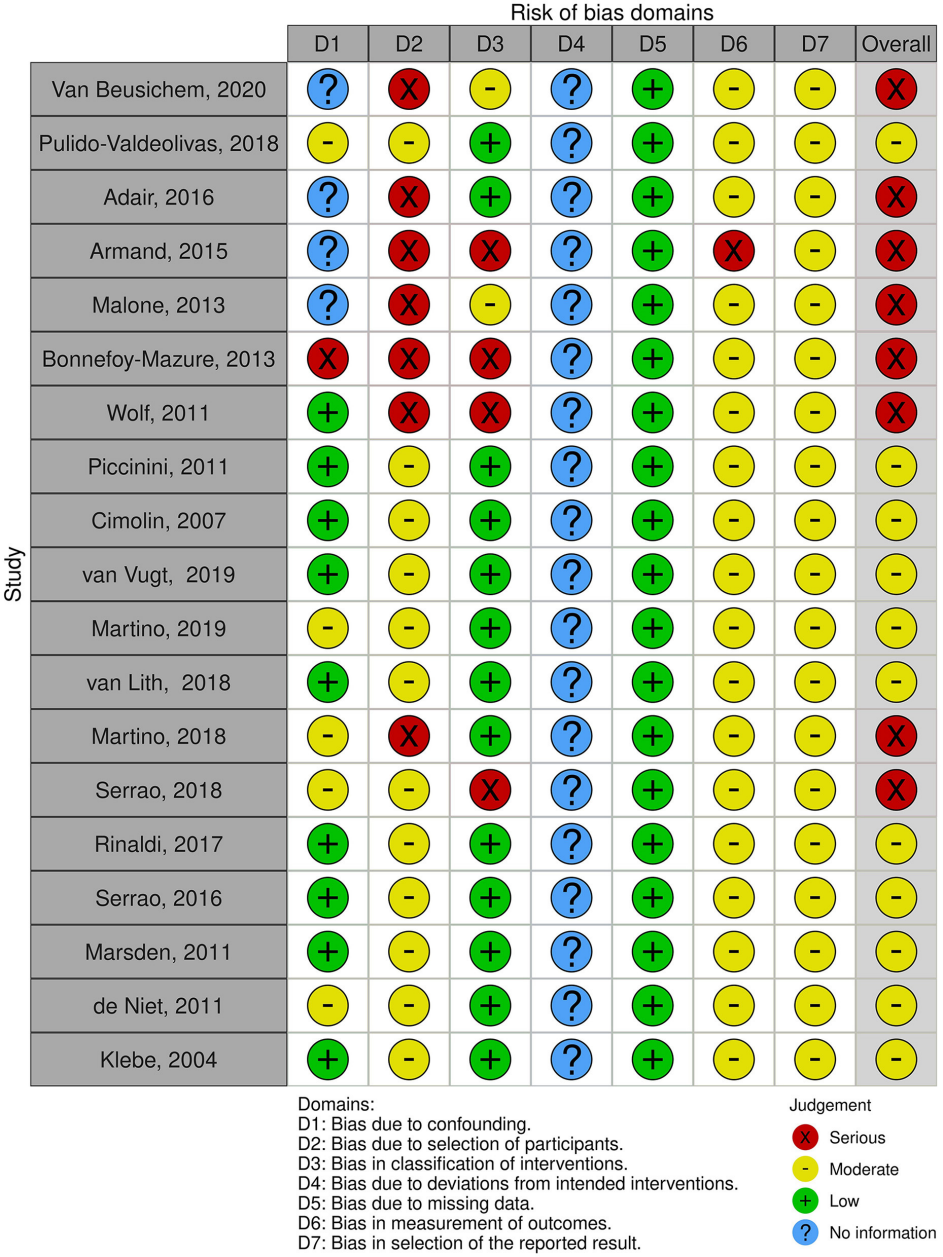
Risk of bias of gait analysis pattern studies: ROBINS-I plot.

**Figure 3:**
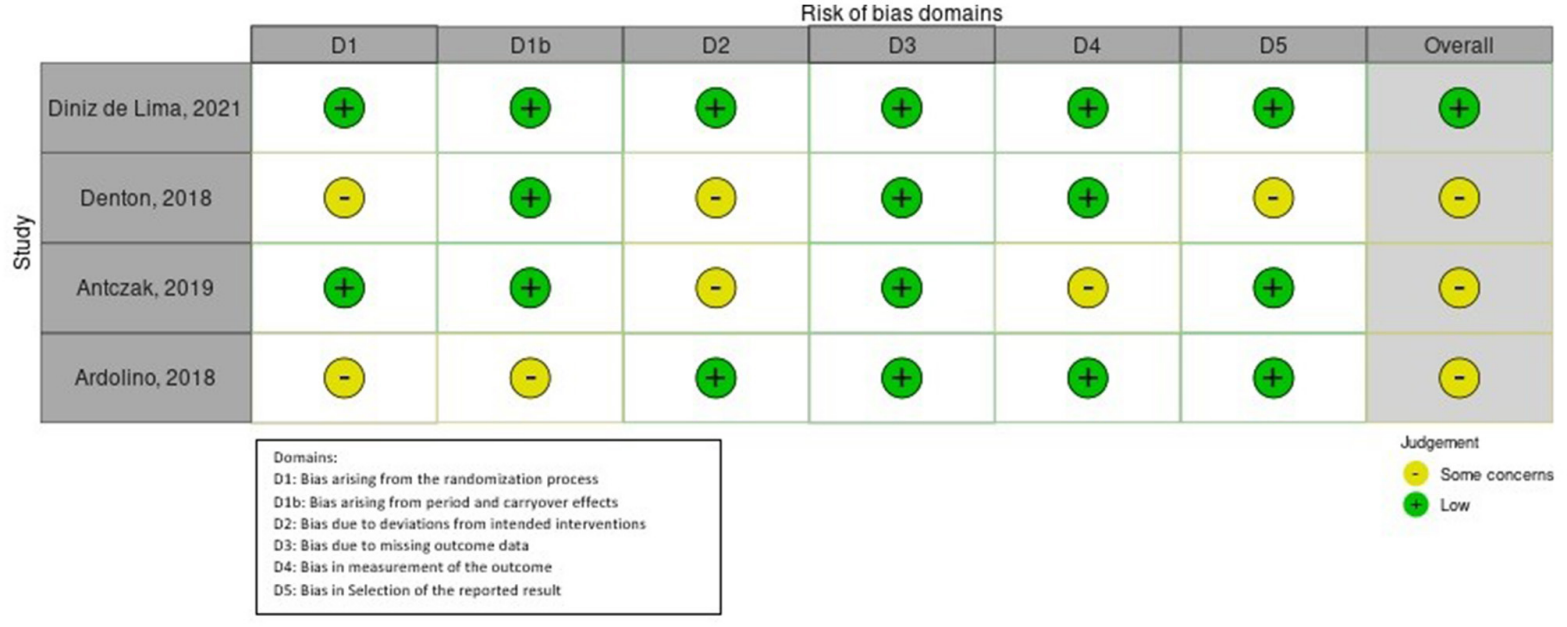
Risk of bias of RCT intervention studies: ROB2 plot.

The results of the quality assessment are represented in Tables 1, 2. An overall synthesis of RoB of included studies is represented in Figures 2, 3. Concerning the confounding factors, age, weight, time from onset, gender, walking abilities and/or aids, and gait analysis protocol were considered relevant to identify and compare gait analysis between groups. For intervention studies, examples of confounding include differences at baseline in patients' characteristics and co-interventions such as drug intake.

**Table 1:** Quality of gait analysis pattern studies assessed by means of the Joanna Briggs Institute (JBI) tools for methodological appraisal of studies.

**Case–control Study/JBI Item**	**Comparability and match**		**Selection and exposure**			**Confounding factors identify and deal with**		**Assessing outcome and exposure length**		**Statistical analysis**	**Overall score**	**Judgment**
	**1**	**2**	**3**	**4**	**5**	**6**	**7**	**8**	**9**	**10**		
Klebe et al. (6)	Y	UN	Y	Y	Y	N	N	Y	Y	Y	7	Fair
Cimolin et al. (34)	Y	UN	UN	Y	Y	N	UN	Y	Y	Y	6	Poor
de Niet et al. (35)	UN	Y	UN	Y	Y	Y	N	Y	Y	Y	7	Fair
Piccinini et al. (36)	Y	Y	Y	Y	Y	N	N	Y	Y	Y	8	Good
Wolf et al. (37)	Y	Y	Y	Y	Y	Y	Y	Y	Y	Y	10	Good
Marsden et al. (38)	Y	Y	Y	Y	Y	N	N	Y	Y	Y	8	Good
Bonnefoy et al. (39)	UN	UN	Y	Y	Y	Y	Y	Y	Y	Y	8	Good
Adair et al. (40)	UN	N	UN	Y	Y	UN	N	Y	Y	UN	4	Poor
Serrao et al. (41)	Y	Y	Y	Y	Y	Y	Y	Y	Y	Y	10	Good
Rinaldi et al. (42)	Y	Y	Y	Y	Y	Y	Y	Y	Y	Y	10	Good
Martino et al. (43)	Y	UN	Y	Y	Y	N	N	Y	Y	Y	7	Fair
Pulido et al. (5)	Y	Y	UN	Y	Y	Y	UN	Y	Y	Y	8	Good
Serrao et al. (44)	Y	Y	Y	Y	Y	Y	Y	Y	Y	Y	10	Good
Van Lith et al. (45)	Y	Y	Y	Y	Y	N	N	Y	Y	Y	8	Good
Martino et al. (46)	UN	Y	UN	Y	Y	Y	Y	Y	Y	Y	8	Good
Van Vugt (70)	Y	Y	UN	Y	Y	N	N	Y	Y	Y	7	Fair
**Case series Study/JBI Item**	**Selection, inclusion and condition measure**					**Information about patients and outcome**				**Statistical analysis**	**Overall score**	
	**1**	**2**	**3**	**4**	**5**	**6**	**7**	**8**	**9**	**10**		
Armand et al. (9)	Y	Y	Y	UN	UN	Y	Y	Y	UN	Y	7	Fair
Van Beusichem et al. (7)	Y	Y	Y	N	N	Y	Y	UN	Y	NA	7	Fair
**Case report study/JBI item** ^*^	**Case reporting and description**							**Takeaway lessons**	**/**	**/**	**Overall score**	
	**1**	**2**	**3**	**4**	**5**	**6**	**7**	**8**	**/**	**/**		
Malone et al. (47)	Y	Y	Y	Y	Y	Y	N	Y	/	/	7	Good

^*^Case report JBI items: 1. demographic characteristics; 2. patient's history; 3. current clinical condition, 4. diagnostic tests or assessment methods and results; 5. intervention(s) or treatment procedure(s); 6. post-intervention clinical condition; 7. adverse events identifications; 8. takeaway lessons.

Y, Yes; N, No; UN, Unknown; NA, Not Appropriate (counted as Y). Studies are listed by year and in the same year by alphabetic order.

**Table 2:** Quality of intervention studies assessed by means of the National Health Institutes (NHI) scale for pre-post non-controlled studies, the Joanna Briggs Institute tools for methodological appraisal of studies (JBI) for case reports and case–control studies, and the Physiotherapy Evidence Database (PEDro) scale for RCTs.

**Pre-post non-controlled**	**1**	**2**	**3**	**4**	**5**	**6**	**7**	**8**	**9**	**10**	**11**	**12**	**TOT**	**Note**	**Judgment**
Klebe et al. (48)	Y	Y	Y	Y	UN	Y	Y	N	Y	Y	Y	NA	9	*n =* 22 pts was judged unclearly in point 5	Good
Rousseaux et al. (49)	Y	Y	N	Y	UN	Y	Y	N	Y	Y	Y	NA	8	Only pure HSP. *n =* 15 pts was judged unclearly in point 5	Fair
Zhang et al. (50)	Y	N	UN	UN	UN	Y	Y	N	Y	Y	N	NA	5	No inclusion criteria and sample description, no multiple time point, and *n =* 11 pts was judged unclearly in point 5	Poor
Bertolucci et al. (51)	Y	Y	N	Y	UN	Y	Y	N	Y	Y	N	NA	7	Only pure genetic HSP. *n =* 13 pts was judged unclearly in point 5. No data in the T2 time point	Fair
de Niet (52)	Y	Y	N	Y	UN	Y	Y	N	Y	Y	Y	NA	8	Only pure HSP. *n =* 16 pts was judged unclearly in point 5	Fair
Denton et al. (53)^*^	Y	Y	Y	Y	UN	Y	Y	N	Y	Y	N	NA	8	For pre-post features; No multiple time point evaluation	Fair
Marvulli et al. (54)	Y	Y	Y	UN	UN	Y	Y	N	Y	UN	Y	NA	7	*n =* 10 pts was judged unclearly in point 5. No clear statistical analysis	Fair
Servelhere et al. (55)	Y	Y	Y	Y	Y	Y	Y	Y	Y	Y	N	NA	10	No multiple time point evaluation, *n =* 33 pts was judged sufficient based on the study by Van Lith 2019	Good
van Lith et al. (56)	Y	Y	N	Y	Y	Y	Y	Y	Y	Y	Y	NA	10	Only pure HSP.	Good
Paparella et al. (57)	Y	Y	Y	Y	UN	Y	Y	N	N	UN	Y	NA	7	Retrospective design. *n =* 18, Missed data > 20% at T3. No clear statistical analysis	Fair
NIH Scale for Pre-post not controlled study: 1. Study question; 2. Eligibility criteria and study pop. clear description; 3. If study participants are representative of populations of interest; 4. All eligible participants were enrolled; 5. Sample size for confidence finding; 6. Intervention is clearly described; 7. Outcome measures are clearly described, valid, and reliable; 8. Blinding of outcome assessors; 9. F-up rate (drop out less than 20% and accounted for in analysis - ITT); 10. Appropriate statistical analysis and p-value report; 11. Multiple time points for outcome measures; 12. Statistical analysis at the group level.															
**Case reports**	**1**	**2**	**3**	**4**	**5**	**6**	**7**	**8**	**TOT**	**Note**	**Judgment**
Pease (58)	Y	Y	Y	Y	Y	Y	N	Y	**7**	No account for adverse events or unanticipated events	Good
Dan et al. (59)	N	N	Y	Y	Y	Y	N	Y	**5**	No account for adverse events or unanticipated events, no description of cases	Fair
Klebe et al. (60)	N	N	N	Y	Y	Y	Y	Y	**5**	No description of the cases	Fair
Molteni et al. (61)	Y	Y	Y	Y	Y	Y	N	Y	**7**	No account for adverse events or unanticipated events	Good
Samuel et al. (62)	Y	Y	Y	Y	Y	Y	N	Y	**7**	No account for adverse events or unanticipated events	Good
Heetla et al. (63)	Y	Y	Y	Y	Y	Y	Y	Y	**8**		Good
Seo et al. (64)	Y	Y	Y	Y	Y	Y	N	Y	**7**	No account for adverse events or unanticipated events	Good
Shin et al. (65)	Y	Y	Y	Y	Y	Y	Y	Y	**8**		Good
Pinto de Souza et al. (8)	Y	Y	Y	Y	Y	Y	Y	Y	**8**		Good
JBI scale for case report: clearly describe 1. Demographic characteristics; 2. Patient's history/timeline; 3. Current clinical condition; 4. Diagnostic tests or assessment methods and results; 5. Intervention(s) or treatment procedure(s); 6. Post-intervention clinical condition; 7. Adverse events identifications; 8. Takeaway lessons											
**Case–control**	**1**	**2**	**3**	**4**	**5**	**6**	**7**	**8**	**9**	**10**	**TOT**	**Note**	**Judgment**
Marsden et al. (38)	Y	Y	Y	UN	NA	N	N	Y	Y	Y	**6**	No identification of confounding factors	Fair
JBI scale for case–control: 1. Groups comparability; 2. Appropriate matching; 3. Same criteria for case and control; 4. Validity of exposure measurement; 5. Equal exposure measurement for both groups; 6. Confounding factors identifying; 7. Confounding factors dealing strategy; 8. Validity of outcome assessment; 9. Length of period of exposure; 10. Appropriate statistical analysis.													
**RCT**	**1**	**2**	**3**	**4**	**5**	**6**	**7**	**8**	**9**	**10**	**11**	**TOT**	**Note**	**Judgment**
Denton et al. (53)^*^	*1*	*1*	*0*	*1*	*0*	*0*	*0*	*1*	*0*	*1*	*1*	***6***	For the randomization part. No blinded study.	Good
Denton et al. (66)	1	1	0	1	0	0	0	1	1	1	1	**7**	No blinded study.	Good
Antczak et al. (67)	1	1	1	0	1	0	1	1	0	0	1	**7**	No baseline comparison, no between-group results	Good
Ardolino et al. (68)	1	0	1	0	1	0	1	1	0	1	1	**7**	Alternating allocation is not a randomization process and no baseline comparison	Good
Diniz de Lima et al. (69)	1	1	1	0	1	1	1	1	1	1	1	**10**	No baseline comparison	Excellent
PEDro Scale for RCT: 1. Specific inclusion criteria; 2. Randomization; 3. Concealed assignation; 4. Baseline comparability; 5. Blinding of patients; 6. Blinding of therapists; 7. Blinding of assessors; 8. Drop out <15% for at least one outcome; 9. Strategy to deal with missing data - Intention to treat; 10. Reported results statistical comparison of at least one outcome; 11. Report of variability data (interval, dev. St.).														

Y: Yes, N: No, UN: Unknown, NA: Not Appropriate (counted as Y), TOT: Total Score. ^*^ Analyzed with both assessment tools (see text). Studies are listed by year and in the same year by alphabetic order.

#### 3.1.1. Quality and rob of gait pattern studies

The quality assessment of GA pattern studies is represented in Table 1. Among the case–control studies, only two studies, namely Adair et al. (40) and Cimolin et al. (34), did not reach a sufficient quality score because of a lack of adequate matching groups and identifying confounding factors. In addition, some concerns were resolved about statistical analysis methods in one of these studies (40), such as the use of discrete variable analysis for continuous variables and the lack of any correction method. The other studies achieved a Good (5, 34, 37–39, 41, 42, 44–47) or Fair (6, 7, 9, 35, 43, 70) quality judgment, associated with a low risk of bias, but presented some limitations. Bonnefoy et al. (39) presented groups different in age, Klebe et al. (6) had some incomplete data (no SIAS marker in 6 patients-50% of the sample, for safety device use), and Wolf et al. (37) declared that comparison of more homogeneous subgroups might be possible but with limited statistical power and the risk of additional bias effects. Some authors (6, 35, 39, 43, 46) did not provide a clear description of the subject characteristics, comparability, matching, or recruitment. Regarding the identification and the management of confounding factors, the authors mostly instructed the control subject to walk at a low comfortable speed (38, 42, 44, 46), to avoid any potential bias due to speed differences between groups and to ensure that the general characteristics of gait could be compared. The subject match was also often done based on age. Some authors (40, 41, 44) did not allow the use of aids to perform gait, resulting in a restriction of the sample size.

The RoB of GA studies was assessed by means of the ROBINS-I tool, as represented in Figure 2. A RoB in D1 resulted whenever the authors did not make appropriate matching between patients and control groups, in particular, considering confounding factors such as age, anthropometric data, or walking speed. For example, Bonnefoy et al. (39) presented the patient's group data including the results of different gait analyses performed by the same group of patients over the years. In the study by Adair et al. (40), the healthy control group's data were derived from another study and the information about comparability was not reported. In case reports and case series studies, no information was provided regarding D1. Only one author performed a blind matching among groups (37). A serious risk in D2 was identified in case reports (47) and case series (6, 9): because of the nature of the studies, the results might affect the recruitment. The risk in D2 was also serious if the patients were retrospectively recruited or excluded (37, 39) or recruited depending on the ability to perform gait analysis (40, 43). Studies based on a retrospective design presented a serious risk in D3. D4 was considered inappropriate in GA pattern studies, because no therapeutic intervention was considered, except gait analysis. All the studies achieved low risk in D5 because they did not report a high relevant percentage of missing data. An overall moderate risk in D6 was evidenced because the methods of gait data analysis were comparable across groups and the outcome measure was probably not influenced by the knowledge of the investigators, nonetheless, no one used any type of blinding method for the analysis. Only the study by Armand et al. (9) presented a serious risk in this domain because the authors compared GA performed with different instruments over the years. An overall moderate RoB was evidenced also in D7 because, in all studies, the outcome measurements and analyses were consistent with the a priori plan, though a pre-registered protocol was never reported. Overall, according to the ROBINS guide flowchart, eight studies presented a serious risk and 11 a moderate risk of bias.

#### 3.1.2. Quality and RoB of intervention studies

The quality of intervention studies is represented in Table 2. NHI scale for pre-post non-controlled studies was used for retrospective observational cohort studies (54, 57) and a randomized pre-post-intervention study (53). JBI case–control scale was used to assess the study by Marsden et al. (71) in which the authors compared the effect of different Functional Electric Stimulation (FES) stimulating patterns between HSP patients and controls providing an inter- and intragroup analysis. Denton et al. (53) randomized the order of presentation of two types of intervention (cooling or warming) in a crossover design and analyzed the pre-post effect, including comparison with controls. Therefore, to avoid bias in reporting results, this study was assessed either with pre-post or with RCT tools for quality assessment.

In the NHI scale, the study questions were clearly stated in the title, abstract, or text. Among pre-post studies, the main limitations were inadequate representation of the population of interest, whenever only pure HSP phenotype was included, small sample size with the lack of power calculation, and no-blind design. Item 12 was considered non-applicable for this type of study population. In the final quality judgment, three studies were Good, six were Fair, and one was Poor.

Among case reports, only two did not reach a sufficient score (59, 60). The most frequent limitation was the lack of declaring the presence or the absence of collateral effects (58, 59, 61, 62, 64); Dan et al. (59) and Klebe et al. (60) did not provide a clear description of the patient's characteristics.

Among RCT (crossover design), one study reached an Excellent rating (10/11) (69) and four studies had a Good quality rating (7/11). Only one study (66) presented a baseline comparison between the crossover groups but did not implement any type of blind procedure. In most of these studies, the operator who administered the treatment was not blinded, whereas the patients and the outcome assessors were blind. In 50% of the studies, the management of missing data was addressed.

Limited to crossover RCTs, the RoB was assessed by means of the ROB2 tool (see Figure 3).

Relative to D1, Denton et al. (66) gave no information about concealed allocation, Ardolino et al. (68) used an alternate allocation design which is considered an incorrect randomization process (72). Some concerns were attributed to the study by Ardolino et al. (68) relative to the D1b domain because the authors did not specify whether the number of participants allocated to each of the two groups corresponded or not, even if they took into account sufficient time to carryover the effects. Denton et al. (66) and Antczak et al. (67) presented RoB in D2 because no information was given regarding the possible influence of unblinding treatment providers on the outcome, even though patients and evaluators were blind. Some concerns in D4 emerged in the study by Antczak et al. (67) because the evaluation of the datasets was not blindly executed. Regarding D5, all the authors referred the study to a pre-specified trial protocol, except for Denton et al. (66). Finally, only Diniz de Lima et al. (69) presented a low RoB.

### 3.2. Evidence synthesis

#### 3.2.1. Gait analysis pattern studies

All patients fulfilled the diagnostic clinical criteria for HSP according to defined criteria (2, 13, 20). The number of included HSP patients ranged across studies from 6 to 50 for the case–control studies and from 1 to 6 for the case series studies. A total of 341 HSP patients were included across all the 19 studies. No explicit differentiation was done among HSP patients with early or late onset—with onset predominantly above or below 35 years according to Harding (13). Nonetheless, nine studies focused on children and young subjects (named median age <18 years old). Most studies considered pure forms of HSP. Very few patients presented complicated forms, including urinary disturbances (6), intellectual deficit, ataxia, and peripheral neuropathy (7).

Characteristics of included studies are represented in Supplementary Table 2.

Since the included studies considered either minors (mostly compared to spastic diplegic subjects) or adults (compared to stroke patients) as samples, the results are presented separately.

#### 3.2.2. Gait analysis pattern studies in children and adolescents

The results of the included studies are represented in Table 3. With sample sizes ranging from 1 to 29, a total population of 111 patients was included in these studies.

**Table 3:** Results in child GA studies.

**Author /design**	**HSP ST results**	**HSP Kinematic results**	**HSP Kinetic results**	**HSP sEMG or other functional results**
Cimolin et al. (34) case–control	HSP showed lower^*^ gait speed, higher^*^ step width, and reduced^*^ anterior step length vs. TD group. Stance time is close to normal and Nsign difference between HSP and SD.	Nsign differences between HSP and SD in pelvis kinematics in all planes. In the sagittal plane: higher ROM of pelvic tilt^*^ and mean pelvic tilt^*^, higher^*^ hip flexion in Gc (angle at iC, min angle in St and max angle in Sw), than TD. Higher knee angle at initial contact than SD^*^ and TD^*^. Higher Knee hyperextension in midstance than TD, similar but longer in the HSP compared to the SD. Higher max ankle angle in stance^*^ and in swing^*^, lower^*^ ankle angle at iC, Lower mean foot progression^*^ than SD. In the transversal plane: normal foot angle, closer to TD.	Nsign statistical difference between HSP and SD. minAP (absorbed power values in early and midstance) close to HC. Lower maxAP^*^ (push-off at terminal stance) than HC.	/
Piccinini et al. (36) case–control	Normal duration of stance phase, shorter^*^ anterior step length, lower^*^ gait speed, and higher^*^ step width when compared to TD group. Nsign differences between HSP and SD.	Frontal and Transversal planes: higher pelvic tilt^*^, pelvic rotation^*^, and pelvic obliquity^*^ than TD. Sagittal plane: Higher^*^ hip flexion during the whole Gc than TD (higher^*^ flex at iC, min hip angle in St and max in Sw). Lower mean hip rotation^*^ than SD. Transversal plane: normal hip, normal foot angle progression. Sagittal plane: higher^*^ knee flexion at iC, quite normal during midstance, and lower^*^ flexion in the swing phase than TD. In addition, 70% of knee hyperextension is in midstance than SD. Than SD: Longer^*^ phase of knee hyperextension during midstance and quite normal position of the ankle during the whole gait cycle. Higher^*^ dorsiflexion of the ankle at iC, St, and Sw than SD	Knee: higher knee flexor moment in midstance than SD and HC. Lower max knee power than SD, close to HC; higher values of minimum knee power. Hip: higher hip moment (max extension moment) and power at iS than HC, Nsign with SD. Ankle joint: lower values of the peak in the plantar flexor moment during tS, quite normal values of minimum absorbed power in iS and midS, and more limited values of maximum ankle power generation at push-off than HC, Nsign with SD.	Low activation of rectus femoris during all gait cycle
Wolf et al. (37) retrospective case–control	HSP group presents Nsign with higher double support and Nsign with lower speed than TD and CP, Nsign difference between HSP and CP	Sagittal plane trunk: HSP had increased^*^ peak trunk tilt velocities vs. CP, quick forward and backward movement of the trunk at the end of loading response and stance–swing transition.	/	/
Bonnefoy-Mazure et al. (39) retrospective case–control	HSP normalized speed is slower^*^ vs. TD and SD groups. Arm swing length is greater^*^ in the top-to-bottom direction vs. SD; significantly greater in the medio-lateral direction compared to the TD group.	Lower Limbs sagittal plane: Nsign differences between the SD and HSP. Differences^*^ between the HSP and HC for HIP, knee, and ankle: Higher hip angle at initial contact^*^, higher minimum hip angle in stance^*^, higher peak of hip angle in swing^*^, and higher mean hip angle in the gait cycle^*^. Higher knee angle at initial contact^*^, higher minimum knee angle in swing^*^, higher mean knee angle in the gait cycle^*^. Lower peak of knee angle in swing^*^ and range of knee angle in the Gc^*^. Higher mean foot progression^*^, Lower peak of ankle angle in swing^*^. Thorax, pelvis, and spine kinematics in the sagittal plane: Higher spine ROM and peak angular velocity than SD. Respect to HC: Higher peak of pelvis angle in stance^*^ and in swing^*^, higher minimum pelvis angle in stance^*^ and in swing^*^, higher pelvis ROM in the Gc^*^, higher mean pelvis angle in the Gc^*^. Respect HC: Lower minimum thorax angle in stance^*^ and in swing^*^, higher thorax ROM in Gc^*^. Respect HC: Higher spine ROM during Gc^*^, Lower mean spine angle in the gait cycle^*^. Elbow and shoulder kinematics: Differences^*^ between HSP and SD groups: lower^*^ peak of shoulder angle, lower^*^ mean elbow angle of flexion, lower^*^ peak of elbow and shoulder angles, lower^*^ minimum elbow and shoulder angles during stance, higher^*^ ratio of the mean angle in the Gc between the left and right sides. Nsign differences between the HSP and HC for the shoulder and the elbow ROM.	/	/
Malone et al. (47) case report	After patella fracture gait speed was slower^*^ compared with the previous analysis	Mild midstance crouch of 20° with reduced knee flexion in swing and dynamic ankle equinus.	Abnormal knee extensor moment in terminal stance	/
Armand et al. (9) retrospective case series	Nsign decreased normalized walking speed in 5 patients (time effect).	/	/	/
Adair et al. (40) case–control	/	The 8/30 parameter distinguishes HSP from HC. In the sagittal plane: HSP had increased excursion of trunk and pelvis ROM; increased posterior trunk lean and increased anterior pelvic tilt. In the coronal plane: HSP had increased trunk ROM obliquity, large peaks of trunk obliquity during the swing phase of the left leg, and delayed maximal pelvic rise. In the transverse plane: HSP had a delay in the timing (later peaks) of maximal posterior pelvic rotation.	/	/
Pulido-Valdeolivas et al. (5) case–control	Six patterns vs. HC: Patterns I, II, and VI: Nsign increased stance times and Nsign decreased single support. Pattern IV, V, and part of Pattern III: reduction of normalized walking speed (Nsign), cadence (^*^), % of cycle in single support (^*^), stance (Nsign), in first double support and second double support (^*^). Normalized walking speed and cadence decrease^*^ with increasing age.	Correlation with age: increased^*^ range of pelvic rotation in tSw, decreased maximum knee flexion vs. HC. Correlation with polyneuropathy: Nsign increased range of pelvic rotation in tSw, increased time to peak of knee flexion, mean hip abduction in first double support and single support, and minimum ankle dorsiflexion in stance. Correlation with GMFCS: GMFCS II and III: delay of peak knee flexion with increased knee flexion at initial contact, increased pelvic rotation, and pelvic obliquity in St. Correlation with thin corpus callosum: Nsign increased ranges of pelvic rotation in second double support and in terminal swing, increased mean pelvic tilt, lower range of ankle dorsiflexion in stance.	/	/
Van Beusichem et al. (7) case series	/	Different parameters on different patterns: Rodda's type III: apparent equinus, and II: jump knee. Rodda's type IV: crouch gait, with increased knee and hip flexion in midstance, with complete foot contact. Type I true equinus; during midstance hyperextension of the knee and complete foot contact.	/	/

ST, spatiotemporal; HC, healthy controls; Nsign, non-significative differences; ^*^, significative differences; tSw, terminal Swing; St, stance; Sw, swing; iS, initial stance; iC, initial contact; midS, midstance; tS, terminal stance; Gc, Gait cycle; pts, patients; SD, spastic diplegia cerebral palsy; TD, typical development; ROM, range of movement; minAP, minimum ankle power; maxAP, maximum ankle power. The studies are ordered by year and within the same year by alphabetic order.

The oldest study by Klebe et al. (6) found a typical gait pattern of patients with sporadic or HSP, compared to healthy subjects, consisting of reduced speed, cadence, and step length; increased step width and increased variation of stride length; reduced sagittal knee range of motion (ROM), with increased minimal knee angle; reduced step height with reduced maximum hip angle; increased maximum ankle angle due to equinovarus feet; and circumduction but no significant variation of foot progression angle.

Van Beusichem et al. (7) applied the Rodda (73) gait classification system for cerebral palsy, to describe the pattern of four subjects affected by a complicated form of HSP due to *de novo* KIF1A mutations. All four gait classes were represented with a progression from classes I and II to III and IV at the last evaluation at 10–18 years.

Pulido-Valdeolivas et al. (5) identified six gait patterns in a group of 26 HSP subjects, aged 4–17 years; the authors compared gait analysis data among patients and healthy subjects, by means of Dynamic Time Warping (5). Pattern I, in the early phase of HSP, was “close to normal” with slightly increased stance time and double support, hip and knee flexion at initial contact (IC), and delayed peak knee flexion in the swing phase. Pattern II presented overall increased anterior pelvic tilt and hip flexion, and increased knee flexion at IC. Pattern III was characterized by knee recurvatum, with reduced and delayed peak knee flexion in the swing phase. Crouch gait corresponded to pattern IV, while constant and severe anterior pelvic tilt, with recurvatum and equinus, distinguished pattern V. Pattern VI was similar to “jump knee” pattern in CP patients (74). Spatiotemporal parameters were relatively spared for patterns I, II, and VI; while they were impaired in patterns III, IV, and V. Asymmetry was described in 27% of HSP subjects, with different patterns in right and left limbs. The authors also found a correlation between GMFCS stages and increased knee flexion at IC, pelvic rotation, and obliquity. They concluded that knee flexion and non-sagittal pelvic movements were relevant indicators of HSP progression. Overlapped polyneuropathy determined an increased range of pelvic rotation in terminal swing and increased time to peak knee flexion, and was most reported in patterns I, II, and III. Abnormal visual evoked potentials (VEPs) were more frequent in subjects classified in pattern III (knee recurvatum).

Armand et al. (9) examined the gait evolution in several subjects affected by HSP (mutations in SPG3A) from the same family. The Gait Deviation Index (75) differed among subjects, but it showed an overall tendency to amelioration from childhood to adolescence and deterioration from adolescence to adulthood.

Adair et al. (40) interestingly analyzed trunk and pelvis kinematics and found increased ROM of the trunk and pelvis in the sagittal plane, with increased posterior trunk lean and anterior pelvic tilt and increased trunk obliquity during the swing phase.

The other four studies included spastic DCP subjects in comparison with HSP and healthy controls (34, 36, 37, 39). HSP and DCP showed similar patterns, and, in both groups, sagittal kinematics could be categorized according to the classification by Sutherland and Davids (74). The principle noticeable difference was that HSP subjects presented more often and longer knee hyperextension during midstance compared to DCP (34, 36, 37). Based on findings by Bonnefoy-Mazure et al. (39), HSP presented significantly reduced gait speed compared to both controls and DCP. Another peculiar characteristic of HSP subjects was increased trunk ROM and peak angular velocity in the sagittal plane during the swing phase (37, 39). HSP presented upper limb patterns similar to healthy subjects, while DCP kept their arms symmetrically elevated, with shoulders abducted and elbows flexed (39).

#### 3.2.3. Gait analysis pattern studies in adults

The results of the included studies are represented in Table 4. The sample size ranged from 6 to 50 subjects. A total of 230 subjects were included in 10 studies; the mean age at the time of GA was 47 years (SD 2.8 years). The mean disease duration among six of these studies (6, 41–44, 46) (in the others no data were available) was 19.7 years, with a prevalence of early-onset forms compared to late-onset forms.

**Table 4:** Results on adult GA pattern studies.

**Author /design**	**HSP ST results**	**HSP Kinematic results**	**HSP Kinetic results**	**HSP sEMG or other functional results**
Klebe et al. (6) case–control	Lower^*^ gait velocity, stride length, and cadence than HC. Increased^*^ step width and variation of the stride length. Nsign in foot angle. The sum score of the MAS correlated^*^ with the velocity, the cadence, the step height, and the step width.	Nsign between SSP and HSP. Reduced^*^ knee ROM, increased^*^ minimal knee angle. Nsign in hip and ankle ROM. Increased maximum ankle ROM (equinovarus foot) and reduced^*^ maximum hip angle, lower^*^ step height, and increased coefficient of variation of the step height.	/	The CMCT was abnormal in 12 patients (delay in 2, reduced amplitude, and a polyphasic pattern in 10). No correlation between the CMCT and the MAS, age, disease duration, or gait abnormality
de Niet et al. (35) case–control	Lower walking speed than HC.	/	/	Increased activity levels during the first half of the St. Greater^*^ MAearly than HC. MLV was relatively constant without distinct peaks in HSP. Lower^*^ MLVmax during the St. Nsign in the proportions of phase shifts observed within the SLR time window, which were low.
Marsden et al. (38) case–control	Slower^*^ normal and maximal walking speed and cadence. Slower standing up/sitting down times and lower scores on the Berg balance scale.	Reduced knee flexion and knee extension in the swing phase, decrease^*^ in peak-to-peak knee amplitude.	During preswing: reduced peak ankle power generation and increased^*^ knee extensor torque. Increased^*^ peak hip flexor power. The reduction in ankle power and the increase in knee extensor torque were associated with a reduction in knee flexor velocity in preswing. Correlations: The ankle power generation was correlated to the isometric ankle plantar flexion strength, and the size of the knee extensor moment was correlated with the degree of passive stiffness in the knee extensors.	/
Serrao et al. (41) case–control	Nsign in mean speed value between groups. Increased^*^ step width and reduced^*^ step length vs. HC. Effect^*^ of patients' subgroup (s): higher^*^ walking speed in s3 than in s1, lower stance duration in s3 than in s1, higher swing duration in both s2 and s3 than in s1, lower second double support duration in s3 than in s1 and higher step length in s3 than in both s1 and s2 and in s2 than in s1.	Three subgroups (s) of patients were identified. s1: reduction^*^ of ROM at hip, knee, and ankle joints; s2: reduced^*^ ROM of knee and ankle joint, but hip joint ROM Nsign than HC; s3: increased^*^ of hip joint ROM, but ankle and knee joint ROM Nsign than HC. Lower^*^ knee and ankle ROM and higher^*^ trunk lateral bending, flexion extension, and rotation ROM and pelvis rotation ROM in patients than in controls. Higher^*^ hip ROM in s2 and s3 than in s1, higher values of knee ROM in s2 and s3 than in s1 and in s3 than in s2, higher values of ankle ROM in s3 than in both s1 and s2 and lower values of pelvis tilt ROM in both s2 and s3 than in s1.	Higher^*^ only knee first and second extensor AI during the stance phase than controls. Lower hip extensor AI during the first double support subphase in s3 than s1.	Higher^*^ values in the TMCf Area of ankle antagonistic muscles (MG-LG vs. TA) than controls (coactivation index). Nsign effect of the subgroup.
Rinaldi et al. (42) case–control	Slower^*^ walking speed. At matched speed higher^*^ values for step width. Nsign in step length, stance duration, and swing duration.	At matched speed: lower^*^ values in knee and ankle ROM. Nsign in hip ROM. Increased CI of both knee and ankle muscles throughout the gc and during the subphases of gait. Positive correlations^*^: between the MAS for both the knee and ankle joints and CI for VL–BF (knee) and TA–SOL (ankle) muscles, respectively. Negative correlation^*^ between the knee CI and walking speed. Nsign partial correlations between the CI and other ST and kinematic parameters. Positive partial correlations^*^ between ankle CI in St and both AWA and APS and between TEC and knee and ankle CI. Negative partial correlations^*^ between R-step and knee and ankle CI. Knee and ankle muscle CI positively correlated with energy consumption and negatively correlated with energy recovery.	Lower^*^ values of AWA and APS (vertical GRF) than HC.	At matched speed, higher^*^ values of CI throughout the gait cycle both for the VL–BF and the TA–SOL pairs of antagonist muscles. Higher^*^ CI in St and Sw for TA–SOL muscles and in the St for the VL–BF muscles, NO diff in the Sw phase for the VL-BF muscles. Energetic parameters: Higher^*^ value of TEC and R-step at matched speed.
Martino et al. (43) case–control	s3: reduction^*^ of walking speed vs. s1, s2 and HC. Reduction^*^ of walking speed in s2 vs. s1. Stance duration is longer in s2 and s3 than in s1 and HC, and shorter stride length in s3 than in s1, s2, and HC. Larger^*^ stride width in s2 than in HC.	Three patient subgroups (s): Increase^*^ of hip joint angle ROM in s1, reduction^*^ of knee and ankle ROM in s2 and s3, and of hip ROM in s3. Inter-subgroup: higher values of ankle joint ROM in s1 than in s2 and s3, higher hip ROM angle in s1 and s2 than in s3, higher knee ROM in s1 and s2 than in s3, and in s1 than in s2. Correlations: with the SPRS score^*^: walking speed, stride length, ankle ROM, knee ROM, FWHM of spinal activation of L2, L3, and L4. ROM of the knee (most sensitive parameter) correlates with FWHM of all segments.	/	Increased^*^ distal leg muscles (TA, PL, SO, MG, LG) and hamstrings (BF and ST) duration of the major bursts in s2 and s3 vs. HC. Trend for the progressive widening of EMGs with the severity of the disease. Higher^*^ coactivation indexes for TA vs. MG-LG in all patient subgroups vs. HC. Mapping in HSP the activity timings in lumbar and sacral segments tend to be quasi-synchronous vs. HC. Maps are characterized by distinct loci of activation of sacral and lumbar segments during late and early stance, respectively. Different^*^ timing of the peak of sacral segments' activity significantly (s3 vs. HC in S2, and s2 and s3 vs. HC and s1 in S1, while Nsign between subgroups).
Serrao et al. (44) case–control	Nsign between groups. Nsign between CA and HSP patients in single and paired ST parameters. Difference^*^ between CA and HSP patients in Mean of step width (triplets and Quadruples of parameters) and Mean, stride-to-stride CV in Quadruples of parameters.	Difference^*^ between CA and HSP patients' ankle ROM (triplets and Quadruples of parameters)	/	/
Van Lith et al. (45) case–control	Nsign differential effect of the SAS between HSP patients and HC in step onset and step length. Nsign effects of the SAS on step length. HSP patients made shorter steps than HC, with and without SAS	/	/	Without SAS: delay in step onset, TA and RF onsets, SOL offset, APA onset compared to HC. SAS accelerated TA and RF onsets in both groups, more in HSP, resulting in near-normal latencies. The SAS accelerated the SO offsets, but greater in HC. The SAS accelerated APA without differential effects between the two groups. APA amplitudes were smaller in HSP patients compared to HC, both with or without SAS. No effect of the SAS on APA amplitudes in either group. The occurrence of the startle reflex in SCM during SAS trials was 64% in HSP patients and 65% in healthy controls, with no difference in TA onset.
Martino et al. (46) case–control	/	Lower^*^ ROM of the knee and ankle joint and lower^*^ foot lift with respect to HC. Smaller^*^ oscillations of the distal segment (shank and foot) (along with smaller ROM in the knee and ankle joints) respect HC. Smaller leg swing and smaller changes in limb length.	/	4 EMG pattern (P) in HSP and HC: Comparable structure of the motor output between the two groups (number of modules and similar synergies, but wider^*^ basic temporal activation patterns P2 and P4 in HSP (FWHM greater^*^ for P2 and P4 in HSP). Correlations^*^: with the SPRS score: shank ROM, foot ROM, FWHM of P2.
Van Vugt et al. (70) case–control	Slower walk velocity, lower cadence, wider^*^ step width, longer step time, and more time spent in the double support phase than HC. Nsign in step length and in single support time between groups.	Higher^*^ lateral trunk flexion than the HC. Nsign in pelvic obliquity between groups. Nsign between-group difference in the AP direction at heel strike or the AP direction at mid-St. Nsign between the groups for the COP-COM separation in the ML direction at heel strike or mid-St. Lower^*^ MOS in the ML direction at heel strike and at mid-St and in AP MOS at mid-St. Nsign in AP MOS at heel strike. Longer^*^ to reach the limits of stability (MOST in the AP direction at heel strike). Nsign in the MOST in the AP direction at mid-St.	/	/

HSP, Hereditary Spastic Paraplegia; SSP, Sporadic Spastic Paraplegia; ST, spatiotemporal parameters; Nsign, non-significative differences; ^*^ significative differences; HC, healthy controls; ROM, range of movement; CMCT, Central motor conduction time (rTMS); FWHM, the full width at half maximum; CI, coactivation Index (antagonist muscles at sEMG); St, stance phase; Sw, swing phase; gc, gait cycle; TA, tibialis anterior; MG, medial gastrocnemius; LG, lateral gastrocnemius; VL, vastus lateral; BF, biceps femoris; SOL, soleus; AWA, area under GRF curve within the weight acceptance; APS, area under GRF curve within the preswing; TEC, total energy consumption; R-step, fraction of mechanical energy (R-step) recovered during each walking step; GRF, Ground reaction force; AP, anterior–posterior direction; ML, medio-lateral direction; MOS, Margin of Stability; MOST, Temporal Margin of Stability; COP, center of pressure; COM, center of mass. SAS, startling acoustic stimulus; RF, rectus femoris; APA, anticipatory postural adjustments; CA, cerebellar ataxia patients; CV, coefficient of variation; AI, angular impulse; TMCf, time-varying multi-muscle coactivation function. MAearly, mean amplitude during the first half of the stance phase; MLV, muscle-lengthening velocity; MLVmax, maximum of MLV. SLR, short-latency stretch response. The studies are ordered by year and within the same year by alphabetic order.

Serrao et al. (41) and Martino et al. (43) identified three kinematic patterns as distinctive of HSP, compared to healthy subjects: increased ROM at the hip, with normal values at knee and ankle; reduced knee and ankle ROM, with normal hip ROM; and reduced ROM at hip, knee, and ankle. A reduced ROM at the knee and ankle was described also in other studies (38, 42, 44, 46), with a decreased foot lift (46), compared to healthy subjects. An increased and premature calf muscle activity was observed both in HSP and stroke subjects, compared to controls, but the contribution of the stretch reflex was excluded (35). Marsden et al. (38) demonstrated that the shorter latency stretch-evoked plantar flexor activity correlated with the increased passive stiffness found at the gastrosoleus in HSP patients, compared to controls. Conversely, no significant difference in knee extensor stiffness was recorded, by comparing HSP and controls. A significant reduction of strength was described, in particular, at the plantar flexors and knee extensors (38). Patterns of coactivation at the electromyography (EMG) were described at dorsi-plantar flexors (41–43) and extensors–flexors of the knee (41, 42). Furthermore, mapping the motor neuron activation in the lumbosacral enlargement of HSP subjects, the activity timings in lumbar and sacral segments tended to be quasi-synchronous because of a progressive widening of the activity involving the sacral segments (43). Conversely, healthy subjects showed distinct loci of activation of sacral and lumbar segments during late and early stance, respectively. Coactivation resulted to correlate with higher energy consumption during gait, based on center of mass (COM) displacements during the gait cycle (42).

Van Vugt et al. (70) analyzed the dynamic postural instability of HSP subjects starting from the distance between the center of pressure and the center of mass (COP-COM separation) to the margin of stability (61) (MOS). The authors found a significantly lower MOS in medio-lateral direction at heel strike and midstance, and in antero-posterior direction at midstance, compared to healthy subjects. van Lith et al. (45) enquired about the anticipatory postural adjustments (APAs) at gait initiation in HSP and controls by studying the StartReact effect. Delayed APAs were observed in HSP subjects, though a starling acoustic stimulus (45) (SAS) positively affected their response, by reducing the activation delay of tibialis anterior (TA) and rectus femoris (RF), close to controls' values. Conversely, the soleus (SO) inhibition was not accelerated upon administration of the SAS.

Finally, lower velocity (35, 42, 43, 70), lower cadence (38, 70), longer double support phase (70), increased step width (41, 42), and increased lateral flexion of the trunk were reported (70). Contrasting data emerged regarding step length and stance duration, being reduced or similar to controls (41–43).

#### 3.2.4. Intervention studies

Included intervention studies focused on adult subjects and no study was found including minors. Population characteristics are summarized in Table 5. Most studies considered pure forms of HSP. Very few patients presented complicated forms, including ataxia, peripheral neuropathy, retinopathy, and epilepsy (53). The methods and results of these studies are reported in Table 6.

**Table 5:** Population characteristics of the intervention studies. Studied are grouped based on the treatment and among the same treatment are ordered by year.

**Interv**.		**Author**	**Population (M) phenotype**	**HSP age Y *mean* ±*SD* (Y range)**	**Population characteristics (gait and functional features to meet inclusion criteria)**
BONT-A + various physiotherapic protocol		Rousseaux et al. (49)	15 pure HSP (10)	48 (25–75% =41–53.5%).	Independent walk with or without assisting devices. 12 pts: extensor gait pattern (knee hyperextension, reduced hip and knee flexion in swing). 3: flexor hip and knee pattern. 9 patients used canes, 2 orthopedic shoes, and 1 ankle–foot orthosis. Spasticity of hip adductors and/or ankle plantar flexors. Difficulties in walking and transfers.
			de Niet (52)	15 pure HSP (12)	47.7 ± 12.3 (20–66)	Community ambulator; bilateral premature calf muscle activity during the loading and/or midstance phase at EMG; balance- and/or gait-related activity limitations in daily life, symptomatic calf muscle spasticity and preserved calf muscle strength.
			Marvulli et al. (54)	10 HSP (7)	40.2 ± 3,6	Paraparetic deambulation with reduced support of back feet, spasticity.
			Servelhere et al. (55)	33 pure HSP (15)	41.7 ± 13.6	With shoes and aid if necessary
			van Lith (56)	25 pure AD HSP (12)	>18	Able to walk > 50 m independently with (adapted) shoes and/or orthoses (but without walking aids) and comfortable gait velocity > 0.4 m/s. Balance- and/or gait-related activity limitations in daily life. Bilateral hip adductor spasticity;
			Paparella et al. (57)	18 HSP (9)	53.9 ± 12.2 (30.7–5.2)	Able to walk with (*n =* 6) or without (*n =* 12) walking aids on a level surface
			Diniz de Lima et al. (69)	55 HSP (36) 41: pure	43 ± 13.4 (19–72)	Able to walk for at least 14 m without stopping. Assistive devices permitted. 22 (60%) walked without device. At least 6 months elapsed since the last injection of Bont-A.
Intrathecal Baclofen (ITB)		Dan et al. (59)	1 pure AD HSP	41	Spastic gait
			Klebe et al. (60)	10 HSP/SSP	Unknown	Unknown
			Molteni et al. (61)	1 HSP	31	Walking impairments, lower limb spasticity, poor balance, nystagmus
			Heetla et al. (63)	1 HSP (1)	49	Able to walk only 100m with assistive devices. Progressive walking difficulties during last 5 years, wheelchair for most activities.
	Stimulation	FES	Pease (58)	1 pure HSP (1)	26	Normal velocity and crouched gait pattern, excessive EMG activity of hamstrings and gastrocnemius. Gait adductor scissoring. Hip flexion contractures. Flexion and extension synergic patterns. Articular impairment because of spastic tone. No strength deficit.
			Marsden et al. (38)	11 HSSP (9) fam.history	57.7 ± 14.2	Able to walk at least 10m with or without a walking aid. Five pts used walking aids. Long-term (>0.5 years) users of FES.
		rTMS	Antczak et al. (67)	9 HSP (7) 7pure/2 compl	40.5	Able to walk 10 meters without or with crutches
		ETOIMS	Shin et al. (56)	1pure HSP (1)	59	Could walk on their own even with the use of an assistive device. Complaining of low back pain. Scissoring, waddling, and feet dragging gait pattern.
		tsDCS	Ardolino et al. (68)	11 HSP (6)	37.3 ± 8.1	Unknown
		SCS	Pinto de Souza et al. (8)	1 HSP (type4)	51	Unable to walk without orthosis
Robot training		Bertolucci et al. (51)	13 pure HSP (6)	46.3 ± 8.9 (31–62)	Able to walk independently for 6 min, with or without walking aids
			Seo et al. (64)	1 pure HSP	28	Walk without assistance using a single cane and bilateral AFO, gradually gait deteriorating. Spastic gait with excessive lumbar lordosis. Bilateral lower limb spasticity and weakness.
	MPH		Klebe et al. (48)	22 SSP/HSP (11)	47.5	Unknown
	Physical therapy		Zhang et al. (50)	9 late-onset HSP	Adult	Unknown
				Denton et al. (53)	22 pure/complHSSP (11)	55 ± 13	Able to walk at least 20 m with (78%) or without a walking aid and have bilateral spasticity in the ankle plantar flexors
			Denton et al. (66)	21 pure/compl HSSP (9)	51.2 ± 12.05	Able to independently walk for at least 20 m with/without a walking aid. The majority (76%) require a walking aid, orthoses, or assistance to walk.
			Samuel et al. (76)	2 pure HSP	45 and 43	1st: exaggerated foot arches bilaterally with typical features of equinovarus deformity. 2nd: bilateral genu recurvatum, equinovarus deformity, and pes cavus with evident toe walking on left

ES, functional electrical stimulation; ETOIMS, electrical twitch obtaining intramuscular stimulation; SCS, spinal cord stimulation; tsDCS, transcutaneous spinal direct current stimulation; rTMS, repetitive transcranial magnetic stimulation; MPH, methylphenidate.

**Table 6:** Details about methods and results of the intervention studies.

**Author and design**	**Sample**	**Treatment and protocol**	**Gait outcome measure**	**Outcome time point**	**Significant improvement results**
Rousseaux et al. (49) pre-post	15 HSP	BoNT-A + usual PT (no in 2 pts) Dose: Botox, different depending on spasticity Site: depending on spasticity SO, GN, TP, FDL, AL, AM	10mWT: step length and w.speed at comfortable and max w.speed (with aids) RMA (leg and trunk), FAC	•Before (d1) and after 2–3 w • After 2–3 m – 5m	W.speed in 10 MWT
de Niet (52) pre-post	15 HSP 10 Ctrl	BoNT-A + home calf stretch (18 ws) Dose: Dysport, 500–750 U dependent on spasticity Site: Triceps Surae, bilateral (electrical stimulation)	10 MWT, Comfortable and max w.speed TUG, BBS, GA parameter	•T0 • T1 (4 w) • T2 (18 w)	Pre-post comfortable w.speed *HSP-Ctr w.speed and balance at T0*
Marvulli et al. (54) pre-post	10 HSP	BoNT-A + PT Dose and site: bilateral with middle dosage of AddM 125 U, GNM e GNL 110U, SO 132 U of Xeomin	Postural and s-t gait parameter	• Before and after 30 d • after 3 m – 4 m - 5 m	W.speed Increase back foot loading
Servelhere et al. (55) pre-post	22 HSP	BoNT-A Dose: Dysport, depending on spasticity Site: leg muscles depending on spasticity	10 MWT, SPRS mFIS (for fatigue)	• Before and after	mFIS (reduction)
van Lith et al. (56) pre-post	25 HSP	BoNT-A + home stretch (10 min 3tpd × 16 w) Dose: Xeomin 150 – 200 U depending on MAS Site: adductors (gracilis, AddM, AddL, palpatory + US)	Gait analysis, w.speed, width in 4.88 m Comfortable and max speed, 6MWT, TUG, Balance (Fall Simulate platform).	• T1: 6 w • T2: 16 w	Gait width, Comfortable w.speed Leg degree in moveable platform
Paparella et al. (57) pre-post	18 HSP	BoNT-A + inpatient intense PT (2 h × 10 times) 2nd treatment injection after 1, 2 y Different dosages of Xeomin, Dysport, Botox Site: depending on spasticity (> HS, RF, GN, ADD)	10 MWT, Comfortable w.speed TUG, WHS, 2mWT, SPRS	• Baseline • 1 m • 3 m	10 MWT, WHS, 2MWT, SPRS (at 1st and 2nd injection) Comfortable w.speed, TUG
Diniz de Lima et al. (69) RCT Crossover	55 HSP	BoNT-A + home PT (1/d × 3 tpw × 8 w) Dose: Prosigne 400U or placebo inj (saline solution) Site: bilateral AddM and TS 100 U (palpatory)	10 MWT, Comfortable and max w.speed SPRS	• T1 (1st inj) - T2 (8 w ± 1 w) • T3 (24–28 w crossover 2nd inj) • T4(8 w)	No significant results
Dan et al. (59) case report	1 HSP *7 Ctrl*	ITB test (75 mcg)	GA on 10MWT at self-selected speed: w.speed, stride length, cadence	• Before test and after 2–4–6h	W.speed; Stride length at 2–4–6h and before vs. ctrl, Cadence at 4–6h
Klebe et al. (60) pre-post	10 HSP	ITB (test and implant + ongoing oral antispastic drugs and PT)	Gait speed, length, width Kinematic parameter on the treadmill (20 s)	• Before • After ITB test (25/50 mcg) • After ITB implant (25 mcg) • After 6 m	w.speed, step length, and step width
Molteni et al. (61) case report	1 HSP	ITB (test 25 mcg and implant)	*Before After test* Time (sec) and N. strides in 10 mWS and 50MWS at self-select max w.speed *Before and After implant* self-select w.speed, step width, stride length, step length (L and R)	• 2 h Before and 3 h after test • Before implant (0 mcg/d) • 6 m (65 m cg/d) • 12 m (85 mcg/d) • 16 m (80 mcg/d) • 24 m (895 mcg/d) after implant	w.speed after test self-select w.speed step length stride length (kinematic parameter no data)
Heetla et al. (63) case report	1 HSP	ITB (continuous test and implant)	Step length, comfortable w.speed Knee flex degrees at IC, LR, MS, TS TUG during the ITB test	• TUG at 0.36–72–108 mcg/d • Before implant • 6 m after implant (105 mcg/d)	TUG, Step length, w.speed Knee flex degree in LR (improved)
Pease (58) case report	1 HSP	FES bilateral (QF, anterior leg mm) 2–3/w × 3 m + home stretching same days	w.speed, cadence, bilateral step length stride length, stance width, time of stance and single limb support (%) during free walking (without stimulation)	• Before • After 7 m	Right hip and knee extension in MS and TS; Symmetry of gait pattern Reduced QF activity in stance (EMGs)
Marsden et al. (38) pre-post	11 HSP 11 Ctrl	Chronic users (2.6 y +- 1.6y) of FES different sequence of stimulation for each pt.	GA in 10MWT: w.speed, max dorsiflex in sw, toe clearance max knee and hip flex PCI (physiological cost index)	• Non-stimulation • After 15 min, Stim bilat on common peroneal • After 15 min, Different Stim	w.speed, toe clearance, dorsiflex in swing
Antczak et al. (67) RCT crossover	15 HSP	rTMS (10 Hz, bilateral 1ary motor area of leg) or sham per 5 time + usual PT (crossover after 1–3 m)	10MWT, TUG	• Before and after 6 w • 2m f-up	No significant results (spasticity reduction at Ashworth)
Shin et al. (65) cohort	1 HSP (5 pts)	ETOIMS (bilateral Q.lomb, mutifidus L4-5, gluteus medius), 2 mA 0,2 ms 1 Hz × 10 s at each point	AMI (Ambulatory Motor Index) 50MWT, w.speed, gait pattern	• Before • Immediately after 1 session	Waddling (reduced)
Ardolino et al. (68) RCT crossover	11 HSP	tsDCS (spinal) anodal or sham. 20 mA, 20 min × 2/die, 5 d/w, At least 3 m. NO PT	5MWT SPRS	• Before and after, 2 m f-up	No significant results (improvement in the anodal group at 5MWT)
Pinto de Souza et al. (8) case report	1 HSP	Chronic Spinal Cord Stimulation implant	GA in 10 MWT: Step length, Step time, Stance and Swing (%), Double limb support (%), Stride length and time, Cadence, w.speed, speed variability (%) SPRS	• 24 m after • ON condition • OFF condition • ON + condition	Lower knee flex-ext muscle torque. Step length (In ON and ON+) Stance%, double limb support% (in OFF), Worsening hip extension in stance, SPRS
Bertolucci et al. (51) pre-post	13 HSP	Lokomat 3/w per 6 w	10MWT, BBS, TUG, 6MWT PCI (O2)	• 1 d before • 3 days after training	10MWT, TUG Decrease cadence, speed, step width, and length, duration of swing phase; Improve left hip extension moment in stance, and hip rotation; worsen pelvic obliquity and left hip abduction during left stance phase
Seo et al. (64) case report	1 HSP	Robot gait training (exoskeleton with partial body weight support) 25 sessions in 6 wks (1/die) + PT 30 min (+30 min)	GA over 8-meter walkway: s-t parameter, kinematic and kinetic hip, knee, and ankle in three planes. 10MWT, 6MWT, FAC, TUG, and BBS	• Baseline and after 6w • 6 m	No significant results
Klebe et al. (48) pre-post	22 HSSP	MPH (methylphenidate) (max 60 mg/day per 6 m)	GA on treadmill: w.speed, cadence, stride length	• Baseline • After 30 min of heating • After 30 min-insulation or not	w.speed between T1-T2 and T3 –T1. No inter groups
Zhang et al. (50) pre-post	9 HSP *Ctrl from database*	Hydrotherapy 45 min, 10 w (group—5 w individual—group—5 w individual)	GA: s-t parameter, kinematics, kinetics	• Pre-post	w. speed, cadence, step length GA pre to post: decrease in the hip, knee, and ankle rotation
Denton et al. (53) RCT pre-post	22 HSSP *19 ctrl*	Warming or cooling worst leg for 30 min (random leg for ctrl)—after 24 h repeat	10MWT max w.speed Foot tapping time	• Before and after • f-up	No significant results
Denton et al. (66) RCT crossover	21 HSSP	Superficial Heating and insulation (30 min or 1 h)—crossover after 24 h.	10MWT, Max w.speed Foot tap time	• Before • Immediately after	*w.speed vs. ctr at baseline* w.speed after worming and (mostly) after cooling as w.speed in ctrl after cooling
Samuel et al. (76) Case report	2 HSP	Intensive PT program (SEIRP: stretching, strengthening, and functional exercise, 60–90 min/d, 6 d/w, per 8 w)	TUG, FRT (Functional Reach Test) 10MWT, 2MWT	• Before and after 4 w • 8 w	10MWT and TUG at 8 w

Studies are grouped based on the treatment and within the same treatment are ordered by year.

Ctr, control; w.speed, walking speed; PT, physiotherapy; US, Ultrasound; w, week; Add, adductor; AddM, adductor magnus; AddL, adductor longus; HS, hamstring; RF, rectus femoris; ADD, adductor; SO, soleus; GNM, medial gastrocnemius; GNL, lateral gastrocnemius; TP, tibialis posterior; TS, triceps surae; FDL, flexor digitorum longus; AL, adductor longus; AM, adductor magnus; s-t, spatiotemporal; 10MWT, 10-m walking test; TUG, Time Up and Go; BBS, Berg Balance Scale; SPRS, Spastic Paraplegia Rating Scale; WHS, Walking Handicap Scale; 2MWT, 2-min walking test; 6MWT, 6-min walking test; FAC, Functional Ambulation Classification; PCI, Physiological Cost Index; 5MWT, 5-min walk test; 50MWT, 50-m walk test; mFIS, modified Fatigue Impact Scale; ITB, Intrathecal baclofen; IC, initial contact; LR, loading response; MS, midstance; TS, terminal stance.

Seven studies researched botulinum toxin injections to reduce spasticity (49, 52, 54–57, 69). Four were cohort prospective studies (49, 52, 55, 56), one was a double-blind randomized crossover study (69), and two were retrospective studies (54, 57). Xeomin (54, 56, 57), Prosigne (69), Dysport (52, 55, 57), and Botox (49, 57) were used (whenever indicated, dilution was 2 to 5 ml). The most frequently injected muscles were gastrocnemius, soleus, adductors magnus and longus, and gracilis (52, 54, 55, 69). One study included tibialis posterior (49). Two studies extended injections to other targets: hamstrings and quadriceps (56, 57); quadratus lumbi, tibialis anterior, flexor digitorum and hallucis, and extensor longus hallucis (56). The sample size ranged from 15 to 55 subjects. A total of 170 subjects were included in the seven studies, of which 98 were male subjects. An overall synthesis of age range was not feasible because data were differently reported as mean, median, or range values, but all patients were over 18 years old. After the injections, self-administered daily stretching (10 min for 2–3 times) (52, 56) or physiotherapy (49, 54, 57) was prescribed. The follow-up ranged from 8 weeks to 5 months (49, 52, 54, 56, 57, 69), only the study by Servelhere et al. (55) did not provide any follow-up assessment. Studies reported a transient reduction of spasticity according to the Modified Ashworth Scale (MAS) and of the injected muscles' strength according to the Medical Research Council (MRC) scale, in the short term. Both receded at 4–5 months follow-up assessment. Furthermore, an increase in range of movement (ROM) within 3 months after injection was reported as increased dorsiflexion, knee flexion, and hip abduction depending on the targeted muscle. Short-term improvement in gait velocity was reported by all studies (49, 52, 54, 56), except Servelhere et al. (55) and De Lima et al. (69). No significant differences were demonstrated at functional tests, except by Paparella et al. (57). This study reported significant improvements at the following tests, 3 months after botulinum and intensive physiotherapy, in 18 subjects: Spastic Paraplegia Rating Scale (SPRS), Walking Handicap Scale (WHS), 10-m walking test (10MWT), 2-min walking test (2MWT), Timed UP and Go test (TUG), the visual analogical scale (VAS), and numeric rating scale (NRS) which assessed the perceived quality of life and pain.

Transient side effects were reported in 19 subjects: muscle strength reduction (52, 55, 69), bruise, transient pain, paresthesia in the site of the injection (69), impairing gait quality (55, 69), sleepiness, and blurred vision in one subject (55). Paparella et al. (57) denied adverse effects.

Four studies researched intrathecal baclofen to reduce spasticity (59–61, 63). Three were case reports (59, 61, 63) and one was a retrospective cohort study (60). Gait analysis at a self-chosen comfortable speed was recorded before and after intrathecal bolus testing (59, 60) or before and after pump implantation. Increased gait velocity and step length were reported by all authors. Klebe et al. (60) described improvement in 5 patients over 10; among them, 2 subjects refused pump implantation because they experienced weakness and unsteadiness. Dan et al. (59) showed that ITB normalized the planar covariation of elevation angles of the thigh, shank, and foot over the gait cycle, thus improving the coordination of the lower limb and reducing mechanical energy expenditure. Heetla et al. (63) reported the reduction of spasticity using MAS, without strength loss and improvement at TUG, which lasted 6 months after implantation. Molteni et al. (61) observed a reduction in the slope of the moment–angle curve of the ankles, which lasted 2 years after pump implantation. The overall baclofen dose range was 25–108 μg, and the overall number of patients involved was 13.

Functional electrical stimulation (71) was enquired by two studies. One case report by Pease et al. (58) reported improvements in gait velocity and knee extension in the stance phase, after FES on the quadriceps and anterior compartment of the leg. Marsden et al. (71) examined a cohort of 11 long-term users of FES (at least 6 months) with and without stimulation and compared them with matched controls. With stimulation (mainly at dorsiflexion, and in some cases also at hip abductors and extensors), an increase in gait velocity and dorsiflexion torque was reported. Long-term follow-up was missing.

One study (65) explored the effect of Electrical Twitch Obtaining Intramuscular Stimulation over the low back and gluteal area, in a mixed population, including one HSP adult. The patient experienced increased speed and reduction of falls and back pain.

Ardolino et al. (68) presented a double-blind, randomized, crossover, and sham-controlled study about anodal transcutaneous spinal Direct Current Stimulation delivered over the thoracic spinal cords (T10–T12). Eleven HSP subjects were involved. They maintained their usual pharmacological treatment but no other intervention (i.e., physiotherapy) was performed during the trial. A significant reduction of spasticity was observed at the Ashworth scale, in particular, at knee extensors and hip flexors, 2 months after treatment. No other functional outcome was improved.

De Souza et al. (8) reported a subject who underwent chronic spinal cord stimulation (SCS) implantation in the posterior epidural space of T11–T12. Alternating ON/OFF phases allowed studying the effect of SCS: improvements in muscle strength and spasticity and at SPRS were reported in ON phases.

Antczak et al. (67) researched the effect of repetitive transcranial magnetic stimulation (rTMS) by means of a blinded, randomized, crossover, and sham-controlled study. Fifteen patients were enrolled, with one dropping out due to a seizure that occurred during a stimulating session. Other adverse effects were headache (several subjects) and sleeplessness (one subject). Usual physiotherapy and oral drugs (59) were provided during the trial. The strength of the proximal and distal muscles of the lower limbs increased, and the spasticity of the proximal muscles decreased. Nonetheless, no functional improvements were observed at the TUG and the 10-m walk test (10MWT).

Two studies (51, 64) researched robot-assisted gait training with partial body weight support: one case report (64) and a cohort study (51) involving 13 pure HSP patients. The treatment lasted 6 weeks. The case report (64) included physiotherapy and overground walking, while the study by Bertolucci et al. (51) provided a gradual reduction of the robotic guidance force and increased workload. An overall improvement in functional tests was observed, with non-significant change in strength, spasticity, and pattern of gait. In the cohort study (51), the improvement was maintained at a 2-month follow-up.

The outcome of an 8-week intensive physiotherapy program including stretching, strengthening, and functional exercise, in two HSP subjects, was described by Samuel et al. (76). The authors reported an improvement in all tests after completing the intervention period: TUG, Functional Reach Test (FRT), 10mWT, and 2mWT.

Zhang et al. (50) researched gait analysis changes after a 10-week hydrotherapy program in 11 HSP subjects. A significant improvement in gait velocity was reported. A significant decrease in the transverse plane rotation of hip, knee, and ankle and an increase in hip and knee peak extension moment were reported.

Klebe et al. (48) performed an open-label study with a longitudinal follow-up at 6 months, in 22 patients treated with 60 mg of methylphenidate per day. Non-significant improvement was observed at gait analysis, MAS, or MRC, at the last assessment. Nausea and sleep disturbances were reported as collateral effects, but only one dropout was recorded, based on worsening of pre-existing urinary disturbances.

Two studies by Denton et al. (53) (randomized treatment with healthy controls) and in Denton et al. (66) (randomized crossover) researched the role of lower limb superficial heating in a total of 43 HSP subjects and 19 controls. The authors demonstrated that heating reduced spasticity, increased dorsiflexor rate of force generation and nerve conduction velocity, and slightly improved gait speed while cooling (53) induced the opposite effects. The application of heating or cooling wrap lasted 30 min.

### 3.3. Meta-analysis

Considering the aim of this review, the meta-analysis was limited to gait and functional outcome measures. Because of the wide variability of the type of interventions and outcomes, and the small number of studies using the same treatment, a meta-analysis was conducted only on studies describing BoNT-A intervention. Two were excluded because the authors did not specify the data results (54) or presented results in terms of median values, which were not comparable with the others (57). Five studies regarding BoNT-A were included. Comparisons were performed regarding the comfortable gait velocity in four (49, 52, 56, 69), the max gait velocity in three (52, 56, 69), the SPRS in two (55, 69), and the TUG results in two (52, 56) studies. In the study by Servelhere et al. (55), the 10mWT was reported as a global value of mean time and SD; then, it was not comparable with other studies, in which the authors reported the gait velocity. In the study by Rousseaux et al. (49), the results were expressed in terms of median values, but it was possible to calculate the mean gait velocity directly from individual raw data. Considering the heterogeneity of time points of evaluations among the studies, data were compared at baseline (t_0_), before 2 months as the first time point (t_1_), and after 2 months as the follow-up time point (t_2_). The analysis was performed estimating the mean and the standard deviation of the change from baseline to each endpoint.

Table 7 summarizes the data used for the meta-analysis for estimating the effect of the BoNT-A; for each selected study, the number of observations (*n*), the mean value, and the standard deviation (*sd*) of the three time points are reported.

**Table 7:** Relevant statistics for the meta-analysis.

**Studies**	**T**_**0**_			**T**_**1**_			**T**_**2**_		
	***n***	***Mean***	***SD***	***n***	***Mean***	***SD***	***n***	***Mean***	***SD***
**Maximum gait velocity**									
de Niet (52)	15	1.33	0.34	15	1.33	0.33	15	1.33	0.37
van Lith et al. (56)	25	1.31	0.41	22	1.33	0.35	22	1.36	0.41
Diniz De Lima et al. (69)	54	1.02	0.57	52	1.01	0.59	n.a.	n.a.	n.a.
**Comfortable gait velocity**									
Rousseaux et al. (49)	15	0.69	0.28	14	0.74	0.24	13	0.68	0.24
de Niet (52)	15	0.90	0.18	15	0.98	0.22	15	1.01	0.19
van Lith et al. (56)	25	0.96	0.25	22	1.04	0.26	22	1.07	0.28
Diniz De Lima et al. (69)	54	0.77	0.38	52	0.74	0.37	n.a.	n.a.	n.a.
**SPRS**									
Servelhere et al. (55)	22	21.60	9.00	22	21.40	9.10	n.a.	n.a.	n.a.
Diniz De Lima et al. (69)	54	16.80	8.25	52	16.40	8.04	n.a.	n.a.	n.a.
**TUG**									
de Niet (52)	22	15	10.4	2.8	15	10.5	2.3	15	10.9
van Lith et al. (56)	54	25	10.6	3.8	22	10.7	4.2	22	10.5

SPRS, Spastic Paraplegia Rating Scale; TUG, Time Up and Go; n, number of patients included in the study at the time point; mean, sd, mean value and standard deviation of the considered outcome measure.

**Figure 4:**
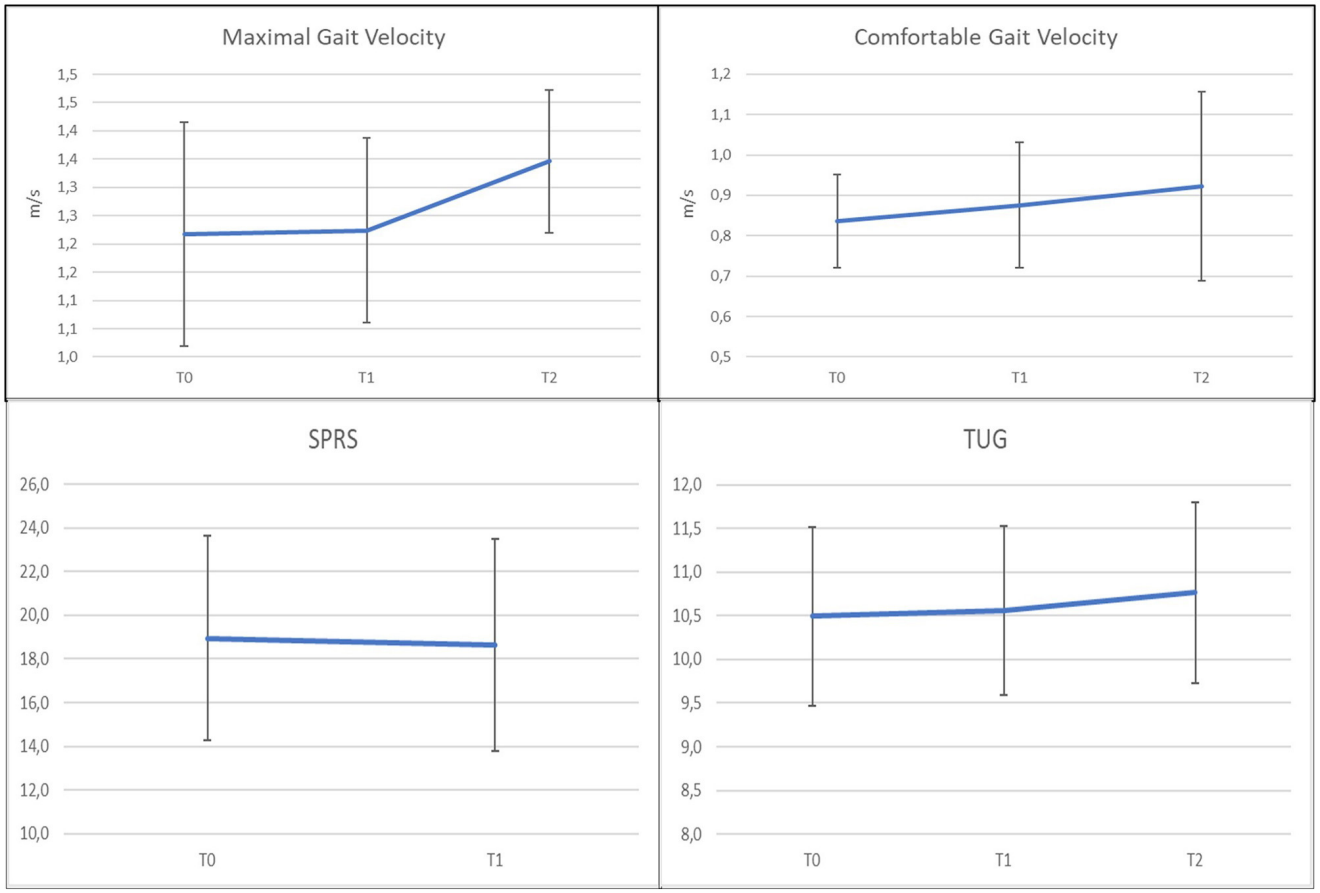
Mean estimates obtained from the meta-analyses relative to Maximal and Comfortable Walking Velocity, SPRS, and TUG, for each examined time period. The error bars represent the standard errors of the estimates of the individual means from the meta-analysis models.

Figure 4 summarizes the weighted means estimates of the various meta-analyses and the relative standard errors, for each time period. An important variability of the estimates for all the considered parameters was generally evidenced. A non-significant effect of BoNT-A was observed in the comparison of the three time periods on the four considered parameters, except for comfortable gait velocity evaluated from t_0_ to t_2_.

**Figure 5:**
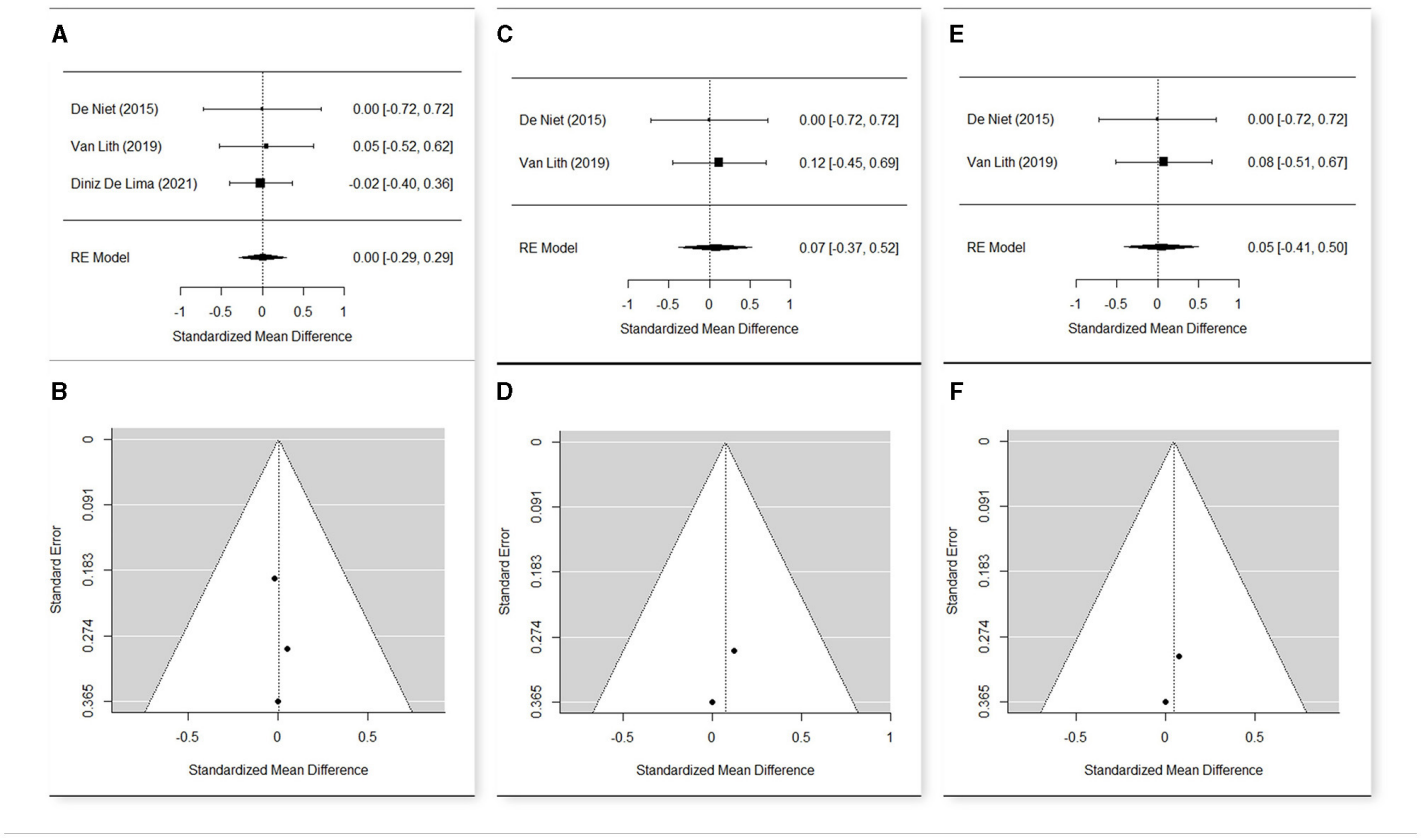
Forest plot **(A)** and funnel plot **(B)** for meta-analysis relative to Maximal Gait Velocity t_1_ vs. t_0_; Forest plot **(C)** and funnel plot **(D)** for meta-analysis relative to Maximal Gait Velocity t_2_ vs. t_0_; Forest plot **(E)** and funnel plot **(F)** for meta-analysis relative to Maximal Gait Velocity t_2_ vs. t_1_.

**Figure 6:**
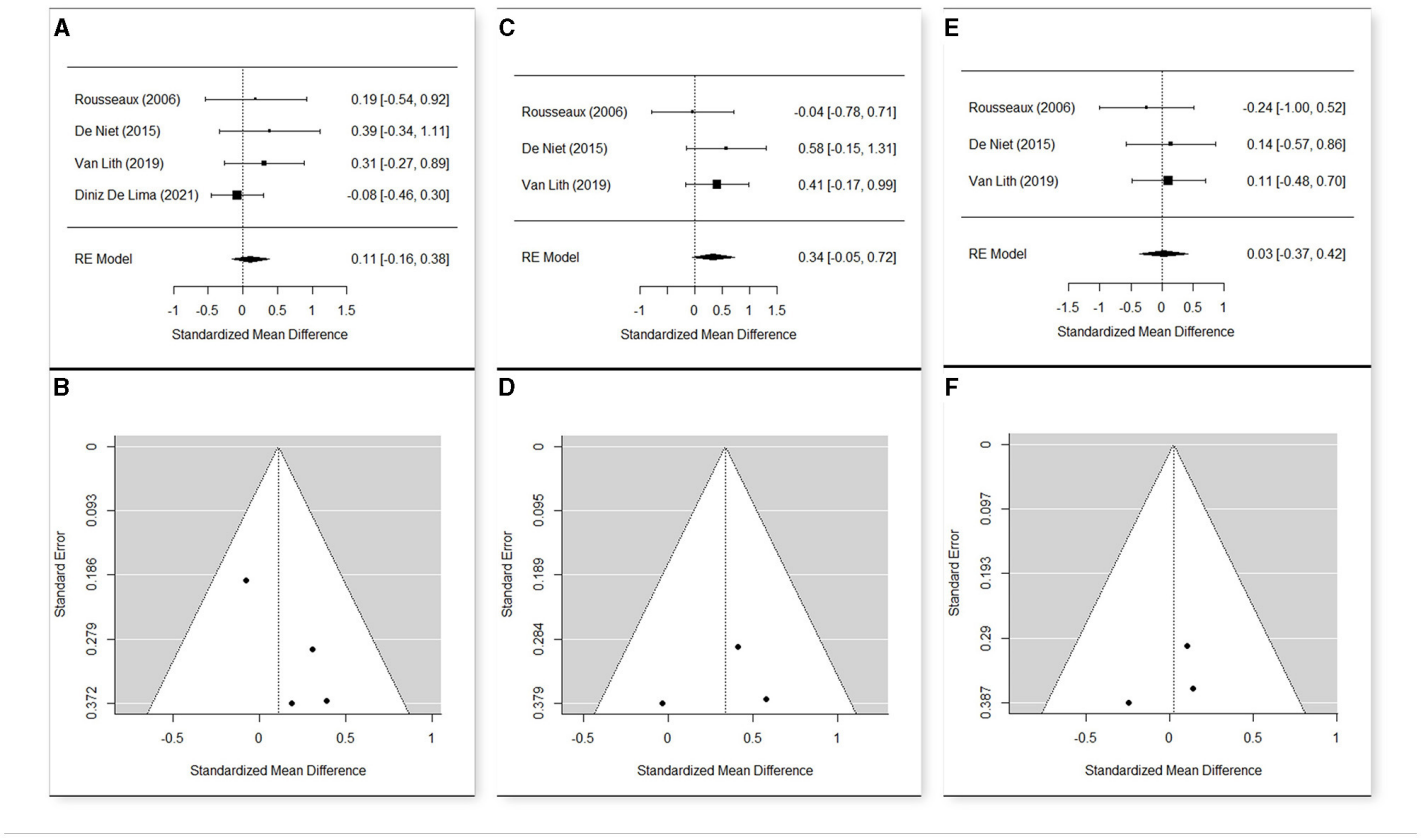
Forest plot **(A)** and funnel plot **(B)** for meta-analysis relative to Comfortable Gait Velocity t_1_ vs. t_0_; Forest plot **(C)** and funnel plot **(D)** for meta-analysis relative to Comfortable Gait Velocity t_2_ vs. t_0_; Forest plot **(E)** and funnel plot **(F)** for meta-analysis relative to Comfortable Gait Velocity t_2_ vs. t_1_.

**Figure 7:**
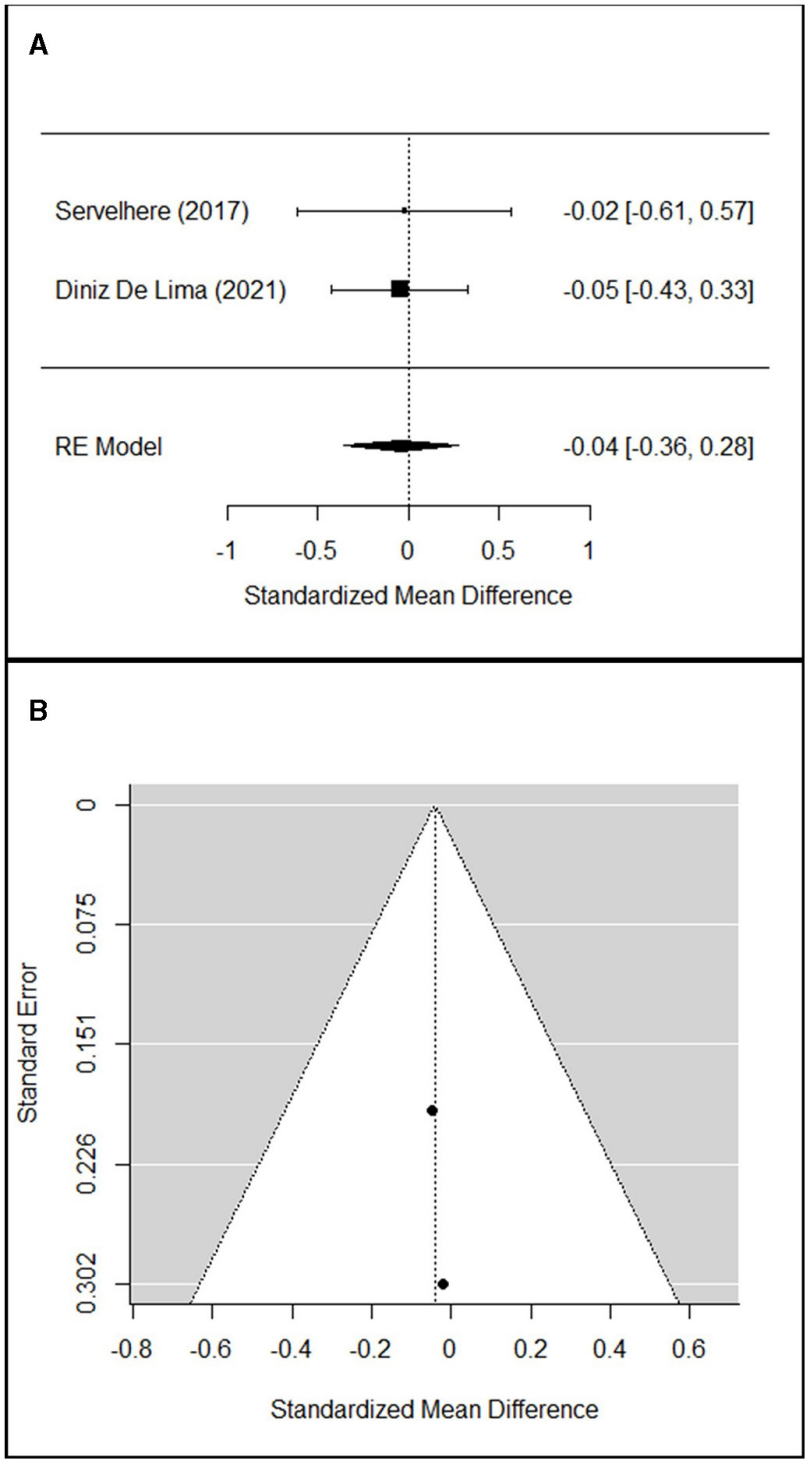
Forest plot **(A)** and funnel plot **(B)** for meta-analysis relative to SPRS t_1_ vs. t_0_.

**Figure 8:**
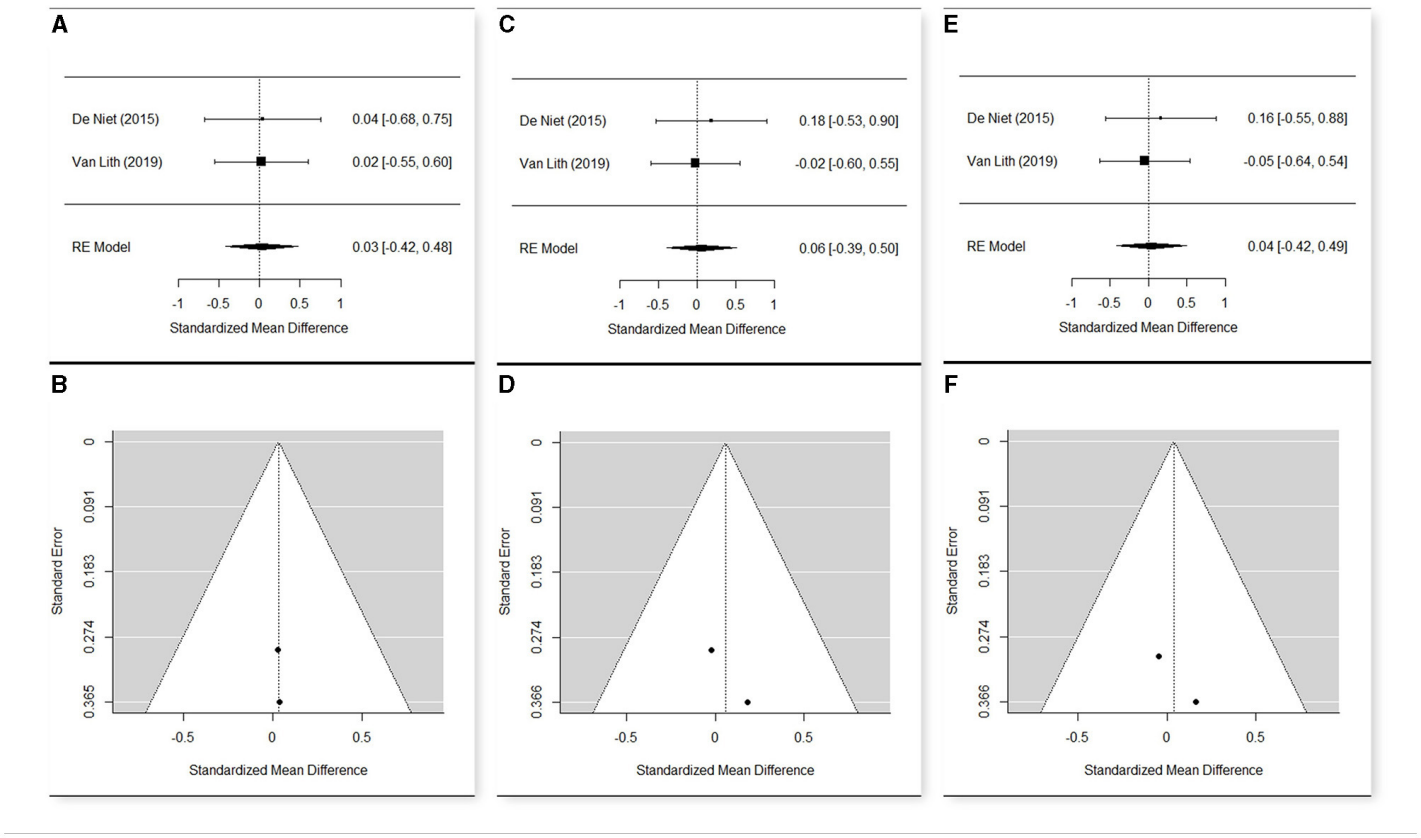
Forest plot **(A)** and funnel plot **(B)** for meta-analysis relative to TUG t_1_ vs. t_0_; Forest plot **(C)** and funnel plot **(D)** for meta-analysis relative to TUG t_2_ vs. t_0_; Forest plot **(E)** and funnel plot **(F)** for meta-analysis relative to TUG t_2_ vs. t_1_.

To estimate the effect of botulinum for each of the outcomes of interest, four independent meta-analyses were performed, as represented in Figures 5–**8** using forest and funnel plots. The forest plot typically summarizes the results of the meta-analysis. The funnel plot shows the estimated treatment effects in terms of standardized mean difference on the x-axis against the standard error (in an inverted scale) on the y-axis. It shows the form of a triangle symmetric to the average treatment effect, with broad variability for small imprecise studies at the bottom of the plot and small dispersion for large, precise studies at the top.

#### 3.3.1. Maximum gait velocity

The meta-analysis (Figures 5A–F) included three studies (52, 56, 69) that presented an overall heterogeneity (*Q* = 9.35, *p* = 0.009 in t_0_; *Q* = 10.40, *p* = 0.005 in t_1_), so a random effect model was used.

The estimated means were 1.217 in t_0_ (*n* = 94; CI [1.019; 1.416]), 1.224 in t_1_ (*n* = 89; CI [1.015; 1.432]), and 1.346 (*n* = 37; CI [1.219; 1.473]) in t_2_.

The change of the maximum gait velocity was not significant from t_0_ to t_1_ (SMD = 0.003; CI [−0.287, +0.293], *p* = 0.983) nor from t_0_ to t_2_ (SMD = 0.073; CI [−0.374, +0.520], *p* = 0.749). Therefore, the change from t_1_ to t_2_ was not significant (SMD = 0.046; CI [−0.409, +0.517], *p* = 0.843).

This non-significance may depend on the great variability of the individual data, but also on the peculiarities of Diniz De Lima et al. (69), in which the average of the maximum gait velocity was considerably lower than that of the other two studies, both in t_0_ and in t_1_. The exclusion of Diniz De Lima et al. (69) from the meta-analysis made the other studies homogeneous (*Q* = 0.05, *p* = 0.817), but the changes in the maximum gait velocity remained non-significant.

#### 3.3.2. Comfortable gait velocity

For the comparison of the comfortable gait velocity, four studies were included (49, 52, 56, 69) in the meta-analysis (Figures 6A–F). A random effects model was used, due to the substantial heterogeneity among the studies (*Q* = 13.26, *p* = 0.004 in t_0_; *Q* = 23.63, *p* < 0.001 in t_1_; *Q* = 21.98, *p* < 0.001 in t_2_). The estimated means were 0.837 in t_0_ (*n* = 109; CI [0.721; 0.953]), 0.876 in t_1_ (*n* = 103; CI [0.721; 1.030]), and 0.922 (*n* = 50; CI [0.688; 1.157]) in t_2_. The mean change was not significant from t_0_ to t_1_ (SMD = 0.108; CI [−0.163; 0.378]; *p* = 0.435), weakly significant in the comparison between the baseline and t_2_ (SMD = 0.335; CI [−0.052; 0.723]; *p* = 0.089), and again not significant t_1_ to t_2_ (SMD = 0.025; CI [−0.365; 0.416]; *p* = 0.898). Also, in this case, Diniz De Lima (69) was peculiar compared to the other studies, because it presented a negative change of the comfortable gait velocity from t_0_ to t_1_ (SMD = −0.08; CI [−0.46; 0.30]). Meta-analysis was then repeated excluding this study. The new results showed a positive estimate of the mean change from t_0_ to t_1_, with a noticeably lower *p*-value, but in any case, non-significant at 95% (SMD = 0.297; CI [−0.086; 0.680]; *p* = 0.129).

#### 3.3.3. Spastic paraplegia rating scale

Only two studies (55, 69) were included in the meta-analysis (Figures 7A, B), and in both cases, no follow-up data were present. Data presented heterogeneity in t_0_ (Q = 4.66, *p* = 0.0308) and also in t_1_ (Q = 504, *p* = 0.0248), so a random effects model was performed. The estimated means of SPRS were 18.948 in t_0_ (n = 76; CI [14, 269; 23.626]) and 18.643 in t_1_ (n = 76; CI [13.769; 23.517]), showing a substantial stability of this parameter (SMD = −0.041; CI [−0.359; 0.277]; *p* = 0.801).

#### 3.3.4. Time up and go

The meta-analysis included two studies (52, 56) (Figures 8A–F) for this parameter that resulted quite homogeneous in the three time points (*Q* = 0.04, *p* = 0.849 in t_0_; *Q* = 0.03, *p* = 0.852 in t_1_; *Q* = 0.13, *p* = 0.721 in t_2_). The estimated means were 10.495 in t_0_ (*n* = 40; CI [9.468; 11.512]), 10.561 in t_1_ (*n* = 37; CI [9.591; 11.531]), and 10.765 (*n* = 37; CI [9.733; 11.802]) in t_2_. Due to the large variability of the data, the mean change was not significant from t_0_ to t_1_ (SMD = 0.0300; CI [−0.417; 0.477]; *p* = 0.896), from t_0_ to t_2_ (SMD = 0.056; CI [−0.391; 0.504]; *p* = 0.804), and from t_1_ to t_2_ (SMD = 0.038; CI [−0.418; 0.494]; *p* = 0.870).

## 4. Discussion

One objective of the review was to provide knowledge concerning the characteristics of gait in HSP subjects, which might reveal specific functional compensations and needs, with the secondary purpose of adequately addressing the treatment strategies.

The frame of HSP gait patterns appeared wide and the severity of symptoms varied either among members of the same family (9) or among different ages (7), given an overall progression over time (5, 9). Relevant indicators of HSP progression were identified, such as knee flexion and non-sagittal pelvic movements (5), reduced ROM at the knee, ankle, and hip (41, 46), which appeared to be associated with coactivation and increased energy consumption (42, 46), decreased foot lift (46), and reduced gait velocity (6, 7, 9, 34, 39, 42–44, 70).

Based on the included studies, pathological gait analysis patterns were described in HSP by comparison with healthy subjects, with attempts to identify clusters of gait patterns either in pediatric (5, 7) or in adult (41, 43) HSP subjects. Nonetheless, most of the GA patterns described appeared similar to DCP and stroke for young HSP patients and adults, respectively. Some authors applied cerebral palsy classifications to categorize HSP sagittal kinematics (7, 37).

Nonetheless, some features distinguished HSP from other similar pathological conditions.

Authors who compared the subgroup of young HSP patients to DCP substantially agreed focusing on the knee kinematics as the most typically involved. Similar to DCP, HSP patients presented stiff knee gait with reduced knee and hip flexion in the swing phase, insufficient knee extension in terminal swing, and insufficient hip extension in stance (5, 39, 40). Nonetheless, longer knee hyperextension was often observed during midstance compared to DCP (5, 34, 36, 37). This may be interpreted as compensation to rectus femoris weakness/hypoactivation (quadriceps avoidance pattern) (34, 36) to achieve a supportive reaction and avoid joint collapse during walking, rather than one manifestation of spasticity, as suggested by EMG pattern (36). While DCP subjects presented higher rectus femoris and hamstring activation, low activation of knee extensors was reported in HSP, with increased absorbed power and decreased generated power at the knee (34, 36). Knee extensor weakness was confirmed at the MRC assessment (36). Based on these observations, ankle–foot orthoses, often recommended in DCP to reduce recurvatum of the knee, might interfere with the HSP knee stabilization strategy (34).

Along with HSP progression, an important increase of knee flexion in midstance and at initial contact was observed, which is similar to the crouch pattern in DCP. This condition might be related to hamstring spasticity/over-activity (5), or most probably to failure of the knee extensor moment (47) because of abnormal quadriceps function, associated with inadequate hip extensor moment, due to weakness of hip extensors (5). Furthermore, a crouch gait pattern was observed following Achilles tendon lengthening surgeries in two HSP patients (39).

Differently from DCP, the ankle joint kinematics, such as the mean foot progression and the global ankle functioning, appeared more or less similar to healthy controls, with global normal foot orientation (34, 36, 39).

An increased anterior pelvic tilt with reduced hip extension appeared to be a typical pattern, explained by iliopsoas muscle spasticity (5) or by hip extensors and hamstring weakness with increased lumbar lordosis (40).

Several studies researched upper body behavior, which is a novelty in GA studies. Increased trunk movements in the sagittal and coronal plane with retroposed trunk and lateral flexion were the most recurrent features. They may be attributed to compensatory patterns to muscle weakness, to assist limb clearance in the swing phase, resembling the “hip abductor avoiding gait” and “hip extensor avoidance gait”, respectively, previously described in people with spina bifida (39, 40). Trunk movements were also characterized by a Double-bump trunk pattern, with twice occurring large peaks of the out-of-phase thorax and pelvis movements, throughout the gait cycle (39, 40). This might be a compensation for distal deficits, related to good control of spinal segments, which is typically maintained in HSP patients (39). Moreover, while DCP patients used synchronized (co-contraction) upper limb and pelvis–thorax movements to increase equilibrium, conversely, HSP patients showed significant and rapid spine tilt, with almost normal shoulder and elbow movements (37, 39). Nonetheless, excessive lateral and posterior trunk movements might also lead to increased energy expenditure, then require the use of mobility devices to help energy conservation and prevent future joint deterioration (40).

Compared to DCP, HSP patients presented a more physiological position of the hip in the transversal plane (36, 40), and this was interpreted as a physiological correction of neonatal femur anteversion in the first years of life in HSP patients, compared to a persistence of this condition in DCP patients, where neuromotor anomalies are present at birth (36). Also, Klebe et al. (6) in an adult cohort denied inward foot rotation.

The stiff knee was also reported as the most common pattern in adults affected by HSP, with a reduced ROM at the knee and ankle (38, 42, 44, 46). Similar to younger patients, these patterns were almost unanimously attributed to weakness, confirmed at MRC, and increased stiffness of plantar flexors and quadriceps, rather than spasticity (35, 38). Furthermore, patterns of EMG coactivation at dorsi-plantar flexors (41–43) and extensors–flexors of the knee (41, 42) were described. They were attributed to decreased cortical inhibition, related to the degeneration of the corticospinal tract, which was not completely compensated by extrapyramidal pathways. One alternative pathway, i.e., the reticulospinal system, was studied by van Lith et al. (45), enquiring the APA after SAS. The authors demonstrated that the reticulospinal pathway might compensate for the corticospinal tract degeneration, accelerating TA and RF activation. Conversely, it failed acceleration of SO inhibition, with a persisting deficit of inhibitory motor control.

Finally, increased step width (6, 41, 42, 44, 70) was often reported and might be interpreted as one strategy to increase stability.

The predominant role of weakness above spasticity was confirmed by the intervention studies. Because of secondary weakness, oral baclofen was mostly withdrawn and only 50% of patients responded to bolus infusion test according to Klebe et al. (60). One case report (59) showed improved lower limb locomotor coordination, speed, stride length, and cadence after ITB bolus. Heetla et al. (63) reported improvement after ITB implantation in terms of gait velocity and spasticity reduction, without strength loss, lasting at a 6-month follow-up. The authors suggested that a continuous infusion test was more effective because it allowed a slight dose increase, reducing adverse effects and providing sufficient time for patients to explore positive outcomes. Regarding studies on botulinum injections, the meta-analysis demonstrated a significant improvement limited to the comparison between the baseline and t_2_ at the comfortable gait velocity, which was not relieved as significant at t_0_-t_1_. This might be attributed to an initial limiting role of post-botulinum weakness (52) that was subsequently overcome. Nonetheless, a significant improvement was not observed at maximum gait velocity. This might be related to an increase in spasticity (even after pharmacological inhibition) and more impaired motor control, associated with augmented velocity. Functional improvements were reported, in particular, in the study by Paparella et al. (57), but botulinum was followed by intensive physiotherapy, which might have contributed to the improvements, based on increased physical activity and reconditioning as observed after gait training. In addition, some concerns should be considered regarding the analysis and the reporting of results in the study by Paparella et al. (57).

A meta-analysis was not possible; however, functional improvements were reported by individual studies after intensive active interventions, such as physical therapy training (including stretching, strengthening, and functional exercises), hydrotherapy, and robotic gait training. Significant or close to significant improvements were reported, in particular, for gait velocity, and, whenever measured, for BBS, TUG, and 10MWT. Only one case report by Seo et al. (64) reported a contradictory reduction of TUG and 6MWT after robot-assisted gait training, while other outcome measures improved (speed, 10MWT, and BBS). The positive rebound of such intensive active interventions might rely on reconditioning through augmented physical activity, as in other pathological conditions (77, 78). Nonetheless, no significant changes in kinematics and kinetics were observed, suggesting that physical activity might improve fitness and the ability to perform compensatory strategies rather than modifying gait patterns. Furthermore, the evidence is limited by a lack of follow-up and very small samples. The benefits might recede following the withdrawal of the training, as it is, for example, in cerebral palsy (77), which has the advantage of being a non-progressive disease. As for the general population, it is advisable to increase physical activity, hopefully integrated into daily life, to maintain or improve fitness, with a possible positive impact on gross motor activities. Nonetheless, this is limited to subjects with sufficient motor skills to be able to undertake training.

Two studies by Denton et al. (53, 66) demonstrated that superficial warming of the legs may reduce spasticity and increase nerve conduction velocity in the very short term, while the opposite effect may be expected by cooling. This confirms previous data (79) supporting the application of such techniques in immediate pre-stretch or pre-exercise periods.

FES (58, 71) determined an improvement limited to gait velocity, but long-term follow-up was missing and samples were small. No improvement was reported at the functional test (SPRS, gait velocity, TUG, 5MWT, and 10MWT) after rTMS (67), tsDCS (68), and ETOIMS (65). Furthermore, sample size and follow-up were very limited. Only one case report (8) researching SCS described improvements at SPRS at 12 months after implantation. Nonetheless, increased difficulty in controlling gait balance and uncomfortable paresthesia was referred by the patient. The authors attributed it to the SCS-induced block of proprioceptive pathways (8). Information about any adverse effects was missing in the other studies, except for rTMS. Antczak et al. (67) reported one case of a seizure occurring during the third session of stimulation, which induced the patient to drop out of the trial. One patient complained of sleeplessness and several subjects reported headaches during the first and second sessions of stimulation, but they all completed the study. Based on the included studies, further evidence is needed to support the role of previous techniques, which might be considered complementary interventions.

### 4.1. Safety and feasibility aspects

Computerized gait analysis is a safe procedure in patients with HSP of any age (no unfavorable events were reported), and only in one study, did six patients require a safety belt suspended from the ceiling of the laboratory, without weight support.

Muscle weakness was the main adverse effect reported following botulinum injections and intrathecal baclofen test. Nonetheless, it resolved at the termination of the pharmacological effect. Minor and uncommon side effects, after botulinum injections, were bruising, transient pain, paresthesia, falls or stumbles, decreased balance confidence (52, 69), slurred speech, handwriting incoordination, and inability to stand up and walk (55). Seizure (one subject) was reported as a major adverse effect following rTMS (67). Minor side effects were sleeplessness (one subject) and headache.

### 4.2. Limitations

One limitation of the present study was not considering the study design as an exclusion criterion, intending to collect as much data as possible in such a rare pathological condition and maintain a more powerful study design. Furthermore, we did not distinguish between the internal validity and statistical analysis validity of the studies, and a statistical analysis of quality score assessment was not performed.

The principal limitation concerning studies researching the GA pattern was that they included only subjects who could walk, without assistive devices, for a sufficient distance to carry out the exam. Therefore, more compromised patients were excluded from the pattern analysis.

Furthermore, limited to young HSP subjects, almost all studies performed the GA once, except Armand et al. (9), and longitudinal information about gait patterns is lacking. Therefore, any possible change related to growth or HSP progression has not been studied.

Relative to intervention studies, several limitations must be underlined: short or lacking follow-up, small samples, wide variability in treatment protocol, and most of all, the absence of studies involving younger HSP subjects.

Finally, most of the included studies, in particular, those researching the GA patterns, were limited to pure forms of HSP. This met the need to select uniform samples and reduce confounders. Nonetheless, a partial representation emerged of the more complex and wide range of HSP clinical phenotypes.

## 5. Conclusion

Knee kinematics and kinetics represent the most peculiar patterns in HSP, compared to DCP and stroke, in particular, related to knee hyperextension in midstance, as compensation to plantar flexor-knee extensor couple deficit.

Other typical patterns are non-sagittal pelvic movements and reduced ROM at the knee, ankle, and hip, which relates to coactivation and increased energy consumption.

Spasticity in HSP hinders muscle weakness, so caution is required while considering interventions to reduce spasticity. Botulinum induced a significant improvement in gait at a comfortable velocity approximately 2–3 months after the injection. This improvement resulted as non-significant immediately after the treatment, probably due to initial weakness.

Limited evidence suggests that intensive physical activity (overground or robot-assisted gait training, functional exercises, and hydrotherapy) and FES might determine improvement in the very short term in gait velocity-related outcomes. Future studies are needed to study the effectiveness of these approaches in HSP subjects.

## Data availability statement

The raw data supporting the conclusions of this article will be made available by the authors, without undue reservation.

## Author contributions

SF: Conceptualization, Investigation, Methodology, Writing – original draft, Writing – review and editing. AC: Investigation, Methodology, Writing – original draft. NF: Data curation, Formal analysis, Methodology, Writing – original draft. GM: Investigation, Writing – original draft. IS: Investigation, Writing – original draft. SS: Supervision, Writing – review and editing.
