# Supplementary material for: Gait analysis patterns and rehabilitative interventions to improve gait in persons with hereditary spastic paraplegia: a systematic review and meta-analysis
Source: Front Neurol. 2023 Sep 20;14:1256392. doi: 10.3389/fneur.2023.1256392 (PMC10548139; doi:10.3389/fneur.2023.1256392)
Supplement: Supplementary file 1 [file Table_1.DOCX]

Supplementary Material

**1 Supplementary table 1.** Search Strategies.

| **Database** | **Searching Strategy** | **n.** |
| --- | --- | --- |
| **Pubmed** | ((“Spastic Paraplegia, Hereditary”[Mesh]) OR (“spastic parap*”[title/abstract]) OR (“hereditary spastic paraparesis”[title/abstract])) AND ((“orthotic devices”[Mesh]) OR (“orthotic devices”[title/abstract]) OR (“orthesis”[title/abstract]) OR (“splints”[Mesh]) OR (“splint*”[title/abstract]) OR (“Botulinum Toxins”[Mesh]) OR (“botulinum toxins”[title/abstract]) OR (“Physical Therapy Modalities”[Mesh]) OR (“[physiotherapy](https://www.ncbi.nlm.nih.gov/portal/utils/pageresolver.fcgi?recordid=608fd6ba580157303d04f8aa)”[title/abstract]) OR (“rehabilitation” [Subheading]) OR (“rehabilitation”[Mesh]) OR (“rehabilitat*”[title/abstract]) OR (“gait”[title/abstract]) OR (“gait analysis”[Mesh]) OR (“gait”[Mesh]) OR ("Walk Test"[Mesh])) AND ((“Gait Disorders, Neurologic”[Mesh]) OR (“Mobility Limitation”[Mesh]) OR (“Recovery of Function”[Mesh]) OR (“walk*”[title/abstract]) OR (“gait improvement”[title/abstract]) OR (“gait”[title/abstract]) OR (“gait”[Mesh]) OR (“gait analysis”[Mesh]) OR ("Walk Test"[Mesh]) OR ("Walking"[Mesh]) OR (“gait pattern”[title/abstract])) | 477 |
| **Cochrane search manager** | Searching:  # 15 ((Hereditary Spastic Paraplegia OR hereditary spastic paraparesis OR spastic parap*)):ti,ab,kw : N 124  # 16 ((rehabilitation OR rehabilitat* OR orthotic devices OR orthesis OR splint* OR botulinum toxin* OR [physiotherapy](https://www.ncbi.nlm.nih.gov/portal/utils/pageresolver.fcgi?recordid=608fd6ba580157303d04f8aa) OR gait OR gait analysis OR Walk Test)):ti,ab,kw : N 78773  # 17 ((Gait Disorders OR Mobility Limitation OR Recovery of Function OR walk* OR gait improvement OR gait OR gait analysis OR Walk Test OR gait pattern)):ti,ab,kw : N 52375  Searching # 18 = ((#15 AND #16 AND #17)) 🡪 N 46 | **46** |
| **RehabData** | Current Search: View Articles, including International Research, containing all of the words: gait, containing the exact phrase: '"Hereditary Spastic parap*"', containing at least one of the word(s): '"gait" OR "walking" OR "rehabilitation" OR "orthotic" OR "rehabilitat*" OR "devices" OR "orthesis" OR "splint" OR "botulinum" OR "toxin" OR "Physical" OR "Therapy" OR "physiotherapy" OR "walk*" OR "test" OR "gait" OR "pattern" OR "speed" OR "analysis" OR "mobility" OR "improvement" OR "recovery" OR "function"' | **2** |
| **PEDro Advanced search** | - **Advanced Search:** Title/abstract: HSP AND gait. - **Advanced Search:** Title/abstract: HSP AND rehabilitation. - **Advanced Search:** Title/abstract: HSP AND orthesis (OR orth*) - **Advanced Search:** Title/abstract: HSP AND Therapy: orthoses, taping, splinting - **Advanced Search** - HSP AND gait training, HSP AND gait impairment, HSP AND gait pattern, HSP AND gait rehabilitation, HSP AND gait speed, HSP AND walking speed, HSP AND walk - **Advanced Search:** Title/abstract: HSP AND gait improvement | **2** |

**2 Supplementary table 2.** Characteristics of included studies concerning gait analysis patterns in subjects affected by HSP.

| **Author /design** | **n. pts (M), median age (y) and phenotype** | **Sample description** | **Control group (M) intervention** | **Experimental GA protocol and instrument** | **Outcome measures** |
| --- | --- | --- | --- | --- | --- |
| Marsden J, et al. 2012 case-control | 20 (12) HSP/SSP  49±13.9y  type 1 | Able to walk at least 100 m with or without a walking aid: 8 patients use walking aids (1 stick n = 6; 2 sticks/crutches n = 2).  Walk without the use of any external electrical stimulation or use of any orthotic.  No regular anti-spasticity medication or BTX-A within the last 3 months. | 18 HC matched for age, gender and height;  Walked at a matched speed and cadence. | Markers placed on standardised bony landmarks and wands (Codamotion, UK).  Customised 10 m walkway containing two embedded force plates.  sEMG on medial GM, TA, RF and medial IC. | ST, Kinetic and Kinematic parameters of hip, knee and ankle joint |
| Klebe S,et al. 2004 case-control | 22 (11) HSP/SSP, 15HSP, 47.5 (range 35-61),  sporadic and familial, early and late, pure and complicated | Able to perform the contraction of the tibialis anterior muscle. | 18 age matched HC performed normal gait analysis | Before GA: gait speed calculation during overground walk 13 m at their own selected confortable speed. GA: three trials of 20 seconds of barefoot walk in motordriven treadmill at personal fait velocity.  Infrared movement analysis system (4 infrared cameras and video processors).  Seven spherical markers to each leg. Treadmill speed was adjusted to the subject’s individual gait velocity measured during normal gait. | ST and Kinematic parameters of hip, knee and ankle joint. 20 consecutive walking cycles were averaged |
| Martino G, et al. 2018 case-control | 35 (23) HSP, results on 29 (20),  48.7y ± 16.1  Pure | Walking independently on a level surface. 3 subgroups based on kinematic behavior during walking: s1: significantly augmented hip ROM. s2: significant reduction in the knee and ankle joints ROM. s3: reduced hip, knee and ankle joints ROM.  Gait disturbances exclusively spastic in nature. | 35 HC (21), results on 30.  Walk at slower speeds | Walk barefoot along a 7-m walkway at comfortable self-selected speeds while looking forward, 15 trials recorded. SMART-D optoelectronic motion analysis system comprising 8 cameras.  Marker on fifth metatarso-phalangeal and lateral malleolus joint, lateral femur epicondyle, greater trochanter and gleno-humeral joint.  sEMG data from 12 right muscles: SO, MG, LG, PL, TA, RF, VM, VL, ST, BF, GM, TFL. Only those strides whose speed was within the range of 1–4.5 km/h were retained here for further analysis | ST and kinematic parameters of hip, knee and ankle joint |
| Martino G, et al. 2019 case-control | 21 (13) HSP  45.7y ± 15.8 | Walk Independently. Gait disturbances that were exclusively spastic. | 20 HC.  Walk at matched walking speeds | Walking at comfortable self-selected speeds along a 7m walkway. 300 Hz - optoelectronic SMART-D motion analysis system, 8 infrared cameras.  8 markers bilaterally (fifth metatarsophalangeal joint (5MT), lateral malleolus, lateral femur epicondyle and greater trochanter).  EMG data from 12 right muscles: MG, LG, TA, PL, SO, RF, VM, VL, ST, long head BF, TFL and GM. | ST and Kinematic Gait parameters. Intersegmental coordination, time-varying elevation angles of limb segment (thigh, shank, and foot). |
| Rinaldi M, et al. 2017 case-control | 23 (17) HSP  49.04 ± 16.31 y  Pure | Able to walk without assistance or walking aids on a level surface.  All patients were undergoing physical therapy (lower limb and stretching exercises, balance, and gait training). Oral antispastic drugs (baclofen or tizanidine) in 5 patients | 23 HC,  walk at low speeds during 15 trials (10 trials  self-selected speed and 5 trials slow walking) separated by 1 min rest  periods. | Walk barefoot at comfortable self-selected speeds along a walkway 10 m in length.  300 Hz with optoelectronic motion analysis system SMART-D System consisting of 8 infrared cameras spaced around the walkway.  22 reflective spherical markers.  Two dynamometric platforms (Kistler 9286B).  Surface EMG signals were recorded using a 16-channel wireless system (FreeEMG300 System) on TA, SO, VL and BF. | ST paramenters.  Kinematic parameters of hip, knee and ankle joint in the sagittal plane.  Kinetic parameters: right vertical GRF, area under GRF curve within the weight acceptance and pre-swing subphases.  sEMG parameters: VL–BF and TA–SOL coactivation values.  CI during stance, swing and the entire gait cycle.  Energy consumption and recovery parameters |
| Van Vugt Y, et al. 2019 case-control | 10 (6) HSP  53.5 ± 11.5 y.  Genetic diagnosis | walking with little or no assistive aid | 10 (6) HC performed normal gait analysis | Walk barefoot at a self-chosen speed along a 10-m walking track.  A minimum of 3 successful trials were obtained for each participant. A static trial was completed to calibration.  Force plates for the COP location. Reflective spheres in upper and lower limbs. Cleveland Clinic marker set. | ST gait parameters, Kinematics parameters of lateral trunk flexion angle, pelvic obliquity angle. Stability parameter (COM-COP distance, AP-ML COM position, AP, ML MOS, MOST). |
| Van Lith BJH, et al. 2018 case-control | 12 (9) HSP  51y (27–71 y)  Pure AD | Only able to walk | 12 (9) aged-matched HC performed normal gait analysis | In front of a box consisting of two blocks with LED: the first represented a warning signal the second an imperative stimulus. Warning periods (1–3.5 s) and inter-trial periods (6–10 s) were variable and random. Pts instructed to stand on the two force plates with their weight equally distributed between the legs. As soon as the imperative visual stimulus was presented, the participants had to start walking as fast as possible and perform at least three steps (one trial), starting with their preferred leg. The participants performed a total of 16 trials; in 25% of these trials a SAS (startling acoustic stimulus) was presented simultaneously with the imperative stimulus. Reflective markers placed at anatomical landmarks on the heel, ankle and toe of both feet. 8-camera system. | Muscle onset latencies for TA and RF and SO offset Reaction times of muscle (de)activation.  For each APA: maximum increase in vertical force under the stepping leg. Step onsets of the heel and toe markers of the stepping leg for each trial.  Step length.  For each trial with a SAS, we determined whether a startle reflex occurred in SCM. |
| Serrao M. et al. 2018 case-control | CA, PD and 31 (19) HSP  48.4 ± 16.3 y  pure | without assistance or walking aids. Ongoing physical therapy. | 65 (34) HC instructed to walk at a low but comfortable speed | Walk barefoot at a comfortable, self-selected speed along a walkway approximately 10 m in length while looking forward. Optoelectronic motion analysis system, 8 camera, 22 passive spherical markers. Two dynamometric platforms. A calibration procedure was executed before the first data capture, few minutes to familiarize, one-minute rest period between each trial to avoid fatigue. At least five trials were recorded for each patient, first trial was rejected. | ST parameters. Kinematic parameters for hip, knee, and ankle joints in the sagittal plane. Gait variability: Mean ad SD of stride duration value during each stride: stride-to-stride coefficient of variation (CV) to evaluate the within-subject variability. |
| Serrao M. et al. 2016 case-control | 50 (30) HSP  47.70 ± 16.06 y  pure with genetic diagnosis in 30 pts. | able to walk without assistance or  walking aids on a level surface. Ongoing oral antispastic drugs and PT | 50 (27) HC, instructed to walk at a low but comfortable speed. Walking speed was matched between groups considering only those control group subjects whose mean walking speed fell within the range identified by patients’ mean walking speed ± SD | 10 trials per patients walking barefoot at a comfortable, self-selected speed along a walkway approximately 10 m in length while looking forward. 1 minute resting. few minutes to familiarize before starting.  The starting position was adjusted with right foot always landed at least on one of the two force platforms in the middle of the pathway. 8 infrared cameras spaced around the walkway, 22 markers, skin sticks or wand, two dynamometric platforms, SEMG (TA, LG, MG, VL, VM, RF, BF, ST). | Difference from HSP and HC; differences between subgroups. ST parameters. Kinematic parameters for hip, knee, and ankle joints (in the sagittal plane), and trunk and pelvis (frontal, sagittal, and transverse plane). Kinetic parameters of ankle, knee and hip- sEMG: co-activation indices of knee and ankle antagonistic muscles. |
| Mark de Niet et al. 2011 case-control | stroke pts and 6 (5) HSP, 45 ± 13 y  heterogeneous | able to walk without assistance or aids | 13 HC performing the same GA, the leg used for HC data analysis was  randomly assigned. | Walk along a 10-m walkway at a self-selected comfortable speed. Data collected in the middle portion of the walkway where subjects walked at a constant velocity. 5 complete gait cycles were recorded for each leg. Bilateral muscle activity recorded by sEMG from GM and SO, 16 reflective markers. | Timing between muscle lengthening velocity (MLV) and muscle activity. Number of gait cycles in which the phase shift was within 40 - 80 ms (short-latency stretch responses, SLRs). For each gait cycle, the highest positive cross-correlation coefficient and the highest positive cross-correlation coefficient within the SLR time window. |
| Pulido-Valdeolivas I. et al., 2018 case-control | 26 (15) HSP,  median age 7 y (range 4-17y) | Walking aids on a level surface. GMFCS I-III and ability to walk 10 meters with or without assistance. | 33 HC school-aged (23) | 15-meter walkway path which allowed five to seven gait cycles per walk at their natural speed, about 8±12 times. Selected up to five left and up to five right gait cycles according to technical quality. Codamotion system, infrared light emitting markers. | ST parameters. Sagittal kinematic parameters form pelvi, hip, knee, ankle. Classification of relevant kinematic and ST parameters to differentiate different gait patterns. Correlation between gait parameters/pattern and clinical features. |
| Adair B, et al., 2016 case-control | 11 (7) HSP  median age 11y 4mo, interquartile range 4y (6-19)  Genetic diagnosis in two pts (SPG3A and  SPG4) | Children needed to be able to walk 10m without assistive  devices during the gait assessment. 10 patients had a heel landing at initial contact, while 5 had a flat foot landing.  Past soft tissue surgery in 1 pt | 28 HC, trunk data were available from 10 of them. | Walked barefoot over a 10m walkway at their self-selected speed. At least five complete trials were captured for each leg: a single representative trial was considered cause of inter-trial variability with no representative derived mean (the first trial with a clean strike on the force plates and minimal marker drop-out). 12 cameras Vicon MX system. 20 markers and additional three trunk markers placed based on the protocol by Davis et al. and the Vicon Plug-In-Gait (PiG) (Vicon Motion Systems, Oxford, UK) model. The sequence proposed by Baker was utilized to define pelvic kinematics | Kinematic parameter in all planes. The GPS and tGPS for trunk parameters. |
| Bonnefoy-Mazure A, et al., 2013 retrospective case-control | 10 (5) HSP  16.7 ± 5.8 y | GMFCS I-II and ability to walk 10 meters with or without assistance.  Past soft tissue surgery in 5 pts | 12 SD (6) 12.3± 4.5 y with same inclusion-exclusion criteria.  17 (9) TD subjects (age 26.2 ± 2.1y)  Perform the same GA. | Walk barefoot at a self-selected speed along a 10-m walkway, data from at least five gait cycles (GCs) were averaged and considered. 12-camera VICON Mx3+; ViconPeak1, Oxford, UK. Passive reflective markers placed according to the Davis protocol and on the trunk as described by Gutierrez et al. | Normalised gait velocity. Sagittal Kinematic parameter of lower limb, thorax, pelvis and spine, elbow and shoulder. Kinematic parameter of frontal plane for shoulder and elbow. Height-normalised arm swing length. |
| Wolf SI, et al., 2011 retrospective case-control | 29 (21) HSP  22.5 y (ranged 5–63 y) | GMFCS I-III. Three pts used walking aid. | 29 CP patients matched in age, sex, and based on gait disturbance.  29 TD subjects match for age and sex | No protocol detail. Data of at least five strides of different trials were averaged. Vicon 370 motion capture before 2001, Vicon 612 system after 2001. | ST parameters. Sagittal Kinematic parameters of pelvis, hip, knee, ankle and trunk. |
| Piccinini L, et al., 2011 case-control | 9 HSP  8.9 ± 3.1 y  sporadic and familial | GMFCS I-II. Able to walk independently without the use of crutches, walkers or braces | 16 SD, type III Rodda, with no previous surgery or other significant treatments for spasticity.  15 TD | Walked barefoot at their self-selected speed along a 10-m walkway, at least five trials were recorded and checked for each child.  Optoelectronic system with passive markers (ELITE2002, BTS, Milan, Italy), 8-channnels surface EMG system (TeleEMG, BTS, Milan, Italy) on rectus femoris according SENIAM recommendations, and a Video system synchronic (BTS, Milan, Italy). Passive markers placed according to the Davis protocol. | ST parameters. Kinematic parameters in all planes for pelvis, hip, knee and ankle, ROM Pelvic Tilt, Obliquity and Rotation indices. Kinetic parameters for hip, knee and ankle joints. EMG Activity and timing, Knee extensor activity in mid or late stance and swing. |
| Cimolin V, et al., 2007 case-control | 15 HSP  10.07 ± 5,56y (ranged 6-15) | Walk independently without aids | 40 SD (aged 4-17), type III Rodda, no previous surgery or other major treament.  20 HC (aged 5-16). | Walk barefoot at self selected speed along 10mt, collecting 5 trials per child. Optoelectronic system with passive markers (ELITE 2002, BTS, Milan, Italy), 2 force platforms (Kistler, CH), video system synchronised (BTS, Milan, Italy). Passive markers placed according to the Davis protocol. | ST parameters. Kinematic parameters in all planes of pelvis, Hip, Knee and ankle in different gait cycle phases. Mean foot progression angle. Kinetics parameters: min and max ankle power. |
| Armand S, et al., 2015 retrospective case series | 6 (2) HSP  9.3 y  AD (SPG3A) mut, early onset (<4y). | able to walk without assistive devices.  GMFCS II, aged from 6y11m to 12y6m at first GA and from 10y2m to 26y8m at last GA. (Bilateral TS BTX-A in 2 pt, ITB implant in 1 pt, soft tissue surgery in 2 pts). | None | Walk at their self-selected speed along a 10-m walkway. Three assessment during f-up. Before 2008: 6-camera motion measurement system: VICON 460; Vicon-Peak®, Oxford, UK. After 2008: 12-camera motion measurement system VICON Mx3þ; ViconPeak®, Oxford, UK. | Walking speed. Kinematic paramerters in all planes from ankle, knee and hip. Foot progression angle. GDI and GVS for each of the nine MAP at first and last GA. |
| Van Beusichem AE et al. 2020 case series | 4 (1) HSP  12.7 y (ranged 10-18y)  with de novo KIF1A mutations. All Complicated forms | Two patients were not able to walk without assistance. 1st: without aid with AFO; 2nd: walking 5 meters with a posterior walker with maximal support of another person. 3rd: with support of a walking frame only for short distances. 4th: ambulant without support. Orthoses needed in 3 pts, orthopaedic shoes in 1 pts. 2 pts underwent PT. | none | 3D Vicon system for GA with Electromyography (EMG). | Sagittal Kinematic paramerters of hip, knee, and ankle (Rodda classification) |
| Malone A et al, 2013 case report | 1 HSP  12y  early onset | GMFCS II. At age 9: toe walking, age 11: crouch gait and AFO, age 12 bilateral sleeve fractures of both patellae, crutches intermittently, treated with immobilization in cast for 4 w. | none | No detail | Gait speed, sagittal kinematic parameters of knee, kinetic parameters of knee. |

*HC: healty controls, BTX-A: Botulinum Toxin –A, GA: Gait Analysis, ST: spatio-temporal, VL: vastus lateral, VM: vastus medialis, TFL: tensor fasciae latae, BF: biceps femori, TA: tibialis anterior, SOL: soleus, CoM: centre of mass, CI: coactivation index, COP: centre of pressure, MOS: Margin of stability, MOST: Temporal Margin of Stability, AD: Autosomal Dominant, APA: Anticipatory Postural Adjustment, CA: cerebellar ataxia, PD: Parkinson Disease, pts: patients, pt: patient, PT: physical therapy, SD: Spastic diplegia, CP: cerebral Palsy, TD: typical development, TS: triceps surae, ITB: intra-tecal Baclofen. GDI: Gait deviation index, GVS: Gait variable scores, MAP: Movement Analysis Profile, GPS: Gait Profile Score.*
